# Concomitant Inhibition and Collaring of Dual-Species Biofilms Formed by *Candida auris* and *Staphylococcus aureus* by Triazole Based Small Molecule Inhibitors

**DOI:** 10.3390/pharmaceutics16121570

**Published:** 2024-12-08

**Authors:** Humaira Parveen, Sayeed Mukhtar, Mona O. Albalawi, Syed Khasim, Aijaz Ahmad, Mohmmad Younus Wani

**Affiliations:** 1 Organic and Medicinal Chemistry Research Laboratory, Department of Chemistry, University of Tabuk, Tabuk 71491, Saudi Arabia; snoor@ut.edu.sa (S.M.); monalbalawi@ut.edu.sa (M.O.A.); 2 Advanced Materials Research Laboratory, Department of Physics, Faculty of Science, University of Tabuk, Tabuk 71491, Saudi Arabia; s.rasool@ut.edu.sa; 3 Department of Clinical Microbiology and Infectious Diseases, Faculty of Health Sciences, School of Pathology, University of the Witwatersrand, Johannesburg 2193, South Africa; aijaz.ahmad@wits.ac.za; 4 Division of Pulmonary, Allergy, Critical Care and Sleep Medicine, Department of Medicine, University of Pittsburgh, Pittsburgh, PA 15213, USA; 5 Department of Chemistry, College of Science, University of Jeddah, Jeddah 21589, Saudi Arabia

**Keywords:** biofilms, drug resistance, triazole, click chemistry, physicochemical properties

## Abstract

Background/Objectives: Biofilm-associated infections, particularly those involving Candida auris and Staphylococcus aureus, pose significant challenges in clinical settings due to their resilience and resistance to conventional treatments. This study aimed to synthesize novel triazole derivatives containing a piperazine ring via click chemistry and evaluate their efficacy in disrupting biofilms formed by these pathogens. Methods: Triazole derivatives were synthesized using click chemistry techniques. The antimicrobial activity of the compounds was tested against planktonic cells of *C. auris* and *S. aureus* in single and dual-species culture conditions. Biofilm disruption efficacy was assessed, alongside the evaluation of physicochemical properties, oral bioavailability potential, and toxicity profiles. Results: The compound T3 demonstrated potent antimicrobial activity against planktonic cells of *C. auris* and *S. aureus* in both single and dual-species cultures. T3 exhibited significant efficacy in reducing microbial viability within biofilms formed by these pathogens. Physicochemical analyses revealed favorable solubility and permeability profiles, supporting its potential for oral bioavailability. Toxicity assessments showed a non-toxic profile, highlighting a promising safety margin for further development. Conclusions: This study underscores the anti-biofilm properties of novel triazole-piperazine derivatives, particularly T3, against single and dual-species biofilms of *C. auris* and *S. aureus*. These findings position T3 as a promising candidate for developing therapies targeting polymicrobial infections and provide a foundation for future research into alternative strategies for combating biofilm-associated infections.

## 1. Introduction

In healthcare settings worldwide, bacteria and fungi silently contribute to alarming patient mortality rates, posing significant challenges to infection management [1,2]. The rise in antibiotic resistance, coupled with the emergence of biofilms as formidable adversaries, has made the battle against infections increasingly complex [3,4]. Polymicrobial infections, caused by the interplay of bacteria, viruses, fungi, and parasites, present a multifaceted challenge that disrupts inflammation and immune responses, often leading to severe and unpredictable clinical outcomes [5]. The high resistance of these infections to antimicrobial drugs exacerbates the situation, resulting in increased morbidity and mortality rates [4,6,7].

*Candida auris* has emerged as a critical pathogen in this landscape, causing outbreaks globally and becoming notorious in healthcare settings [8]. Its formidable resistance to multiple drugs, coupled with frequent misidentification and an alarming ability to persist in hospital settings, has made *C. auris* a major concern [1,9]. Studies indicate that *C. auris* can linger on environmental surfaces, such as bedside tables and mattresses, and its biofilms demonstrate troubling resistance to common disinfectants like povidone iodine, chlorhexidine, and hydrogen peroxide [5]. *C. auris* biofilms are characterized by unique clustering behavior and exhibit resilience greater than that of biofilms formed by *Candida albicans* and *Candida glabrata* [5,10]. Furthermore, *C. auris* interacts with other skin microbiota, including *Staphylococcus* species, which have their own colonization strategies. *Staphylococcus* species thrive by exploiting skin urea and producing proteases to access nutrients, often overshadowing other skin flora, with *Staphylococcus aureus* being a prominent offender in wound infections [11,12]. *C. albicans* is well-documented for its interactions with skin bacteria, leading to enhanced biofilm formation and increased pathogenicity due to elevated production of adhesins and extracellular enzymes [13,14]. Similarly, *C. auris* is known for its ability to adhere to both biotic and abiotic surfaces, forming biofilms resistant to conventional treatments, including caspofungin, which is typically effective against *Candida* biofilms [10]. Previous research has highlighted the protective role of the *C. albicans* extracellular matrix in shielding *S. aureus* from vancomycin [15]. Recent studies have investigated dual-species biofilms of *C. auris* and *S. aureus* or *S. epidermidis*, examining their impact on resistance to common antiseptics [16]. Although data on *C. auris* interactions with other microbiota remain limited, it is plausible that *C. auris* interacts with *S. aureus*, potentially exacerbating virulence, and complicating treatment regimens.

**Figure 1:**
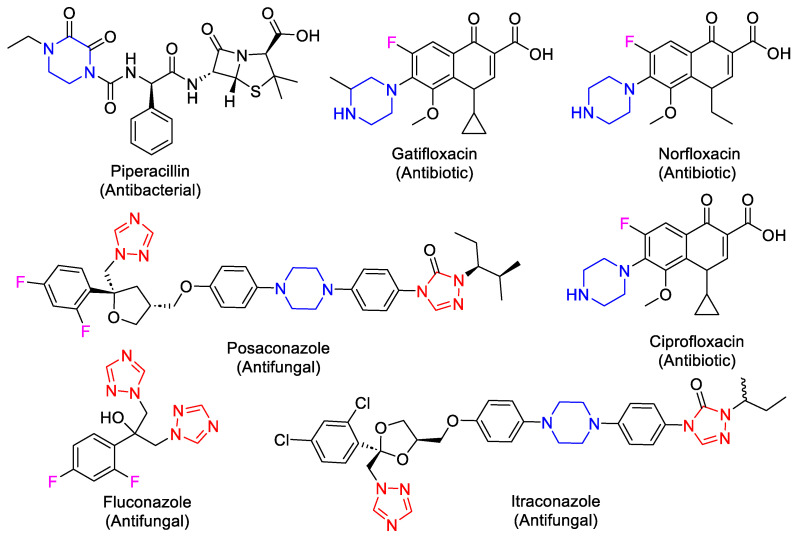
Different antimicrobial drugs featuring a triazole (red) and piperazine ring (blue).

Considering the escalating threat of biofilm-related infections, developing innovative treatment strategies is essential. Disrupting or eradicating biofilms is crucial to overcoming the resilience of microbial communities. Among the various strategies that are being pursued [17,18,19,20,21,22], a promising approaches is the development of small molecule inhibitors designed to penetrate biofilms and eliminate embedded microbes [3,23,24,25,26]. Triazole ring-containing compounds have demonstrated significant antimicrobial properties, particularly against fungi [27,28,29]. Key antifungal drugs such as fluconazole, ketoconazole, itraconazole, and Posaconazole derive their efficacy from the triazole ring structure. Additionally, piperazine, a versatile scaffold found in multiple generations of antibiotics, offers unique advantages in enhancing physicochemical properties, including solubility and permeability [30,31]. As illustrated in Figure 1, incorporating a piperazine ring significantly alters the pKa of a molecule, optimizing its pharmacokinetic profile and improving its water solubility. By leveraging the synergistic benefits of piperazine and triazole through advanced click chemistry techniques and in continuation of our work on the development of new small molecule antimicrobial agents [32,33,34], we have synthesized novel triazole derivatives featuring a piperazine ring. This study focuses on assessing the efficacy of these compounds in disrupting biofilms while also evaluating their physicochemical properties and toxicity profiles. Our goal is to advance our arsenal against resilient biofilm infections. This innovative approach holds promise for creating more effective treatments and improving patient outcomes in the ongoing fight against biofilm-associated infections.

## 2. Materials and Methods

### 2.1. Chemistry

The chemical reagents and solvents were procured from Sigma-Aldrich (St. Louis, MO, USA) and Merck (Darmstadt, Germany). TLC plates used were precoated aluminum sheets (silica gel 60 F_254_, Merck Germany) and visualization was conducted by UV light in a UV cabinet. Heraeus Vario EL III analyser (Hanau, Germany) was used for elemental analysis. Bruker ALPHA FT-IR spectrometer (Eco-ATR, Karlsruhe, Germany) was used for FTIR analysis. Bruker AVANCE 400 spectrometer (Karlsruhe, Germany) (400 MHz) was used for ^1^H and ^13^C NMR spectra using DMSO-*d*_6_/CDCl_3_ as solvent with TMS (Tetramethylsilane) as standard. ESI-MS positive ion mode was recorded on Micromass Quattro II triple quadrapole mass spectrometer (Manchester, UK).

#### 2.1.1. Click Synthesis of 2-(4-((4-(2-Fluorophenyl)piperazin-1-yl)methyl)-1*H*-1,2,3-triazol-1-yl)acetyl Chloride (**3**)

Compound **3** (2-(4-((4-(2-fluorophenyl)piperazin-1-yl)methyl)-1*H*-1,2,3-triazol-1-yl)acetyl chloride) was obtained through a click chemistry reaction of (1-(prop-2-yn-1-yl)-1H-indole) with 2-azidoacetyl chloride in equimolar ratio in DMF. 1-(2-fluorophenyl)-4-(prop-2-yn-1-yl)piperazine (**2**) in turn was obtained by treating 1-(2-fluorophenyl)piperazine (**1**) (1 g, 8.53 mmol) in dry acetone with propargyl bromide dropwise (0.65 mL, 8.53 mmol) at room temperature, resulting in precipitation of the product in acetone in no time as discussed previously [32]. The product was filtered, washed, dried, and used as such in the next reaction.

#### 2.1.2. Synthesis of Quinoline Based 1,2,3-Triazole Derivatives (T1–T12)

**Scheme 1:**
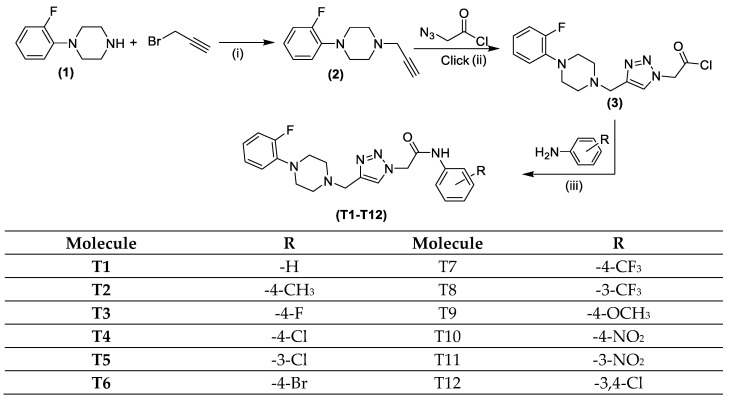
Synthesis of piperazine based triazole acetamide derivatives (T1–T12). Reagents and conditions. (i) Dry acetone, r.t (ii) Sodium ascorbate, CuSO_4_, DMF (iii) HATU/DIPEA, DMF, 60 °C.

The target derivatives (T1–T12) were synthesized following a straightforward synthetic route as shown in Scheme 1 following our previously published protocol [32]. Briefly, compound **3** on treatment with different amines in DMF using HATU/DIPEA at 60 °C resulted the formation of target derivatives (T1–T12). Minimum amount of DMF (5–10 mL) was used as a solvent and the crude compounds were obtained by vacuum evaporating any excess DMF and precipitation in water. The crude compounds were recrystallized twice in dichloromethane: methanol solvent mixture. The physical and spectroscopic characterization data of all the derivatives (T1–T12) is given in the Supporting Information.

### 2.2. Pharmacokinetic Studies/ADMET Profile

Pharmacokinetic and ADMET properties were assessed using pkCSM, an online resource (https://biosig.lab.uq.edu.au/pkcsm/ accessed on 1 July 2024) provided by Cambridge University to evaluate the drug-like potential of the freshly prepared compounds [35]. Additionally, ADMETLab 3.0 (https://admetlab3.scbdd.com/, accessed on 1 July 2024) [36] and SwissADME SwissADME (http://www.swissadme.ch/, accessed on 1 July 2024) [37], an online tool from Swiss Institute of Bioinformatics were used to validate the results. ProTox-3.0 online web tool (https://tox.charite.de/protox3/, accessed on 5 July 2024) was employed to evaluate the toxicity profiles of the most potent derivative T3 along with the reference drug Fluconazole [38]. Additionally, CytoSafe (http://cytosafe.labmol.com.br/, accessed on 5 July 2024) and BeeToxAI (https://beetoxai.labmol.com.br/, accessed on 5 July 2024) which are advanced machine learning based QSAR tools designed by LabMol (http://www.labmol.com.br/, Accessed on 5 July 2024) to predict the acute toxicity of chemicals were used to predict the toxicity of the compounds.

### 2.3. Biological Studies

#### 2.3.1. Fungal and Bacterial Strains Used in Study

*Candida auris* MRL6057 and *Staphylococcus aureus* ATCC29213 were stored as glycerol stock at −80 °C. *C. auris* MRL6057 and *S. aureus* ATCC29213 were revived on sabouraud dextrose agar (SDA; Sigma-Aldrich, St. Louis, MO, USA) and Luria Bertoni (LB; Sigma-Aldrich, USA) agar plates, respectively. *C. auris* MRL6057 was incubated at 35 ± 2 °C for 48 h, whereas *S. aureus* was grown at 35 ± 2 °C for 24 h. Single colonies of *C. auris* MRL6057 and *S. aureus* ATCC29213 were picked up from the freshy streaked plates and inoculated in 10 mL Sabouraud dextrose broth (SDB; Sigma-Aldrich, USA) and LB broth medium, respectively, and incubated in an incubator shaker at 35 ± 2 °C, 200 rpm for 18 h. Thereafter, the cultures were precipitated and washed twice with phosphate-buffered saline (PBS; Sigma-Aldrich, USA) and further adjusted to a concentration of 0.5 McFarland standard (*C. auris*, 1 × 10^6^ cells/mL; *S. aureus*, 1 × 10^8^ cells/mL) using turbidimeter. Prior to experiment the pathogens were diluted to assay-specific concentration in the required media.

#### 2.3.2. Antifungal and Antibacterial Activity Against Individual Pathogen

To prepare the yeast inoculum, the isolates grown on SDA at 37 °C for 24 h were set at a final concentration of about 0.5–2.5 × 10^3^ cells/mL in the sterile saline. The antimicrobial activity of the compounds T1–T12 against individual pathogens (*C. auris* MRL6057 and *S. aureus* ATCC29213) was determined in terms of minimum inhibitory concentration (MIC) and the protocol was adopted from Clinical and Laboratory Standards Institute (CLSI) guidelines [39,40]. A stock solution (10 mg/mL) was prepared in 1% dimethyl sulfoxide (DMSO; Sigma-Aldrich, USA) and the concentration range tested against the individual as well as mixed pathogens was 2500–1.22 µg/mL; experiment was conducted in a 96—well microtiter plate (flat bottom). The included controls were amphotericin B (positive control for yeast), azithromycin (positive control for bacteria), cells growing in presence of 1% DMSO (negative control) and cell free (sterility) controls. The MIC was defined as the lowest concentration of the compound that resulted in the inhibition of visible growth of both test microbes. The results were calculated as a median of three independent experiments.

To verify the microbicidal activity of T3, 20 µL was taken from each well exceeding the MIC, plated onto SDA, and incubated for 24 h at 35 ± 2 °C. The minimum fungicidal/bactericidal concentration (MFC/MBC) was defined as the lowest concentration that resulted in no visible microbial growth on the agar plates [41].

#### 2.3.3. Antifungal and Antibacterial Activity Against Mixed Pathogen

In the quest of discovering a potent antimicrobial agent that can act against yeast as well as bacteria under mixed culture conditions, we evaluated antimicrobial activity of the compounds against mixed culture condition (*C. auris* MRL6057 + *S. aureus* ATCC29213) [42]. Concisely, a 100 µL aliquot of each compound (T1–T12) was combined with 50 µL of yeast and 50 µL of bacterial inoculum in a 96-well microtiter plate and incubated at 35 ± 2 °C for 24 h. MICs were determined as described, followed by MFC/MBC calculations for mixed cultures.

#### 2.3.4. Anti-Biofilm Activity Against Single—And Mixed Biofilms

Compound T3 showed very promising antimicrobial activity against individual as well as mixed culture condition and therefore, it was taken forward for advance study. The inhibitory activity of T3 against single or dual species biofilm was measured at two different time points—biofilm formation and 24 h mature biofilms. The protocol was adopted from previous work [16] with modification. The yeast and bacterial inoculum were prepared at 1 × 10⁶ CFU/mL in RPMI 1640-MOPS, buffered with 0.165 M 3-(*N*-morpholino)propane sulfonic acid (RPMI). The single species biofilm was established in a 96-well polystyrene flat-bottomed microplate by addition of 100 µL aliquot of single microorganism in the wells containing 100 µL of medium, whereas the dual—species biofilm was formed by dispensing 100 µL of *C. auris* and 100 µL *S. aureus* together in the same well. The microtiter plate was incubated at 35 ± 2 °C with gentle shaking (100 rpm) for 90 min to allow cell adhesion. Unattached cells were then removed by washing the wells with PBS, followed by the addition of 100 µL of RPMI to each well. The plates were further incubated at 35 ± 2 °C with shaking (100 rpm) for 24 h to promote biofilm formation. Negative controls (T3-free) and sterility controls were included in the experiment. To evaluate the anti-biofilm activity, 100 µL of the respective medium containing T3 was added to designated wells after the adhesion phase, and plates were incubated under biofilm-forming conditions.

A parallel experiment was conducted to assess the anti-biofilm activity of the test compound on mature biofilms. The biofilms were initially incubated for 24 h, after which non-adherent cells were removed. The remaining sessile cells were then treated with T3 and incubated for another 24 h at 35 ± 2 °C with shaking at 100 rpm. Furthermore, to evaluate the anti-biofilm activity of T3, the metabolic activity of biofilms was checked by using XTT reduction assay by using the protocol published previously [43]. The percent inhibition for single and dual—species biofilms was calculated by using below formula and the minimum dosage were there more than 90% inhibition was recorded was termed as BIC_90_. The experiment was performed in duplicates and was repeated three times.

(1) Percent inhibition=100−(treated O.D.×100/untreated O.D.)

#### 2.3.5. Impact on Microbial Viability

The effect on fungal and bacterial cell viability in single and dual—species biofilms was determined by calculating colony forming units (CFU/mL) [43]. Briefly, following T3 treatment, the attached biofilms were rinsed twice with PBS, scraped, and plated onto SDA plates containing 50 µg/mL chloramphenicol for *C. auris* growth and Mueller Hinton Agar (MHA; Sigma-Aldrich, USA) supplemented with amphotericin B for *S. aureus* growth. The plates were incubated at 35 ± 2 °C for 24 h and colonies were counted on a colony counter and the mean log CFU/mL value was calculate [43].

#### 2.3.6. Impact on Total Biomass Quantification

The effect of T3 on the total biomass (extracellular polymeric matrix (EPM) + sessile cells) was determined quantitatively by violet crystal (VC) staining method [43]. Concisely, after the incubation time was over the biofilms were gently washed with methanol and stained with VC (0.1%) for 15 min. Later, the well was again washed with PBS and air dried for some time. The quantification was performed by adding 33% glacial acetic acid to all the wells, and the optical density (O.D.) was measured at 590 nm using a SpectraMax iD3 multi-mode microplate reader (Molecular Devices, San Jose, CA, USA).

Additionally, a preliminary assay was conducted to visualize the effect of T3 on preformed biofilms. The biofilms were grown on coverslips, treated with T3 following the standard protocol, and then stained with 0.4% VC for 10 min. After washing away the excess stain and air drying the coverslips, the biofilms were examined under a Leica DM 500 light microscope, and images were captured using an attached digital camera.

#### 2.3.7. T3 Extricates Dual—Species Biofilms Formed by *C. auris* and *S. aureus*

The anti-biofilm nature of T3 against 24 h mature single and dual—species biofilms was confirmed with the help of confocal laser scanning microscopy (CLSM; Carl Zeiss, Inc., White Plains, NY, USA) by using the method was described elsewhere [41]. Single and dual-species biofilms were grown on glass coverslips in 24-well microtiter plates under standard biofilm-forming conditions. After 24 h of treatment, mature biofilms were stained with the fluorescent dye FUN-1 (Invitrogen, Thermo Fisher Scientific, ZA, Waltham, MA, USA; excitation at 543 nm and emission at 560 nm) and the Concanavalin A (ConA)-Alexa Fluor 488 conjugate (Invitrogen, Thermo Fisher Scientific, ZA; excitation at 488 nm and emission at 505 nm). Following incubation, the stained coverslips were examined using a Zeiss Laser Scanning Confocal Microscope (LSM) 780 with Airyscan (Carl Zeiss, Inc.). Confocal images of the red (FUN-1) and green (ConA) fluorescence were captured simultaneously in multitrack mode.

#### 2.3.8. Scanning Electron Microscopy (SEM) Analysis

SEM was used to further advocated the biofilm inhibitory potential of T3 against mature biofilm formed by single and dual—species. As described in previous section, 24 h mature single and dual—species biofilm was formed and treated with T3, also the negative control was taken into consideration. The biofilm was fixed with 5% glutaraldehyde and gradually dehydrated using increasing concentrations of ethanol (20%, 40%, 60%, 80%, and 100%). It was then immersed in hexamethyldisilazane (HMDS) and left to dry overnight at room temperature. The coverslips were mounted onto aluminum stubs, carbon-coated, and examined under a scanning electron microscope (SEM; Zeiss Gemini 2 Crossbeam 540 FEG SEM).

#### 2.3.9. Hemolytic Assay for T3

The hemolytic activity was assessed following a previously described protocol [44]. Briefly, horse blood suspension was treated with T3 at concentrations of 0.25× MIC, 0.5× MIC, MIC, and 2× MIC for 24 h at 37 °C. The samples were centrifuged at 2000 rpm for 10 min, and the supernatant was transferred to a fresh tube. The absorbance was measured spectrophotometrically at 450 nm. The blood suspension treated with Triton X-100 (1%) and sterile PBS was used as positive and negative controls, respectively. The following equation was applied to calculate the percentage of hemolysis:

(2) $$\%\;haemolysis=\left[\frac{A450\;of\;test\;compound\;treated\;sample-A450\;of\;buffer\;treated\;sample}{A450\;of\;1\%Triton\;X-100\;treated\;sample-A450\;of\;buffer\;treated\;sample}\right]\times 100\%$$

#### 2.3.10. Statistical Analysis

Each experiment was performed in triplicate, and the results were analyzed using Student’s *t*-test with GraphPad Prism version 9.1.0. A *p*-value of ≤0.01 was considered statistically significant.

## 3. Results and Discussion

### 3.1. Chemistry

The synthesis pathway for the target derivatives (T1–T12) is depicted in Scheme 1. The key compound, 3 (2-(4-((4-(2-fluorophenyl)piperazin-1-yl)methyl)-1*H*-1,2,3-triazol-1-yl)acetyl chloride), was synthesized through a Copper-Catalyzed Azide-Alkyne Cycloaddition (CuAAC) reaction. This involved the reaction of 1-(2-fluorophenyl)-4-(prop-2-yn-1-yl)piperazine with 2-azidoacetyl chloride in a 1:1 molar ratio in DMF, which is known for its ability to facilitate efficient cycloaddition reactions. The precursor, 1-(2-fluorophenyl)-4-(prop-2-yn-1-yl)piperazine (2), was synthesized by reacting 1-(2-fluorophenyl)piperazine (1) with propargyl bromide.

The target derivatives (T1–T12) were subsequently synthesized via amide coupling reactions between various substituted phenylamines and the acyl group of the triazole derivative (3). The yields of these reactions ranged from 80% to 85%, demonstrating the efficiency of the synthetic strategy employed. The use of click chemistry, particularly the CuAAC method, is recognized for its reliability and eco-friendliness, allowing for high yields and purity of 1,2,3-triazole derivatives, which are highly regarded in medicinal chemistry for their diverse biological activities [45,46]. The structures of all synthesized derivatives were confirmed using a range of physical and spectroscopic techniques. The structure of the triazole (3) was validated through elemental analysis, FTIR, ^1^H NMR, ^13^CNMR, and ESI MS. Notably, the FTIR spectrum exhibited characteristic stretching bands at 3185 cm^−1^ (C-H triazole), 3087 cm^−1^ (C-H, Ar), and 1725 cm^−1^ (C=O), confirming the presence of functional groups indicative of the triazole derivative. Importantly, the absence of azide (N≡N at 2100–2160 cm^−1^) and alkyne (2100–2140 cm^−1^) stretching bands confirmed the successful cyclization of 1-(2-fluorophenyl)-4-(prop-2-yn-1-yl)piperazine with 2-azidoacetyl chloride.

The formation of the triazole ring was further corroborated by the characteristic absorption band in the FTIR spectrum between 3185 and 3180 cm^−1^, corresponding to the =C-H stretching of the triazole ring in the target derivatives. In the ^1^H NMR spectrum, a singlet peak at δ 7.76–7.78 ppm (1H, s, triazole ring) and the disappearance of the terminal acetylene proton signal in the aliphatic region further substantiated the presence of the triazole moiety. The ^13^C NMR spectrum showed peaks at δ 143.8 and 123.3 ppm, corresponding to the carbon atoms in the triazole ring, thereby confirming the structure of compound 3. Additionally, the positive ion mass spectrum displayed a peak at *m*/*z* 338.11 [M + H]^+^, providing further structural confirmation. The structures of the target compounds (T1–T12) were established similarly. The formation of the amide bonds in these derivatives was confirmed by characteristic stretching frequencies for C=O and N-H (amide I and amide II bands) observed in the FTIR spectra, which are typical for secondary amides. The consistency of the ^1^H NMR, ^13^C NMR, and ESI MS spectra with the proposed structures of the newly synthesized molecules was detailed in the experimental section and Supporting Information.

### 3.2. Physicochemical Properties

#### 3.2.1. Pharmacokinetic Studies/ADMET Profile

In drug discovery, understanding the physicochemical properties of molecules is critical for determining their potential as effective therapeutics. These properties influence key aspects such as absorption, distribution, metabolism, excretion, and toxicity (ADMET), making their comprehensive evaluation critical for assessing drug-like characteristics. In this study, we conducted a thorough evaluation of the pharmacokinetic and ADMET properties using a combination of advanced tools like pkCSM (https://biosig.lab.uq.edu.au/pkcsm/, accessed on 1 July 2024) [35], ADMETLab 3.0 (https://admetlab3.scbdd.com/, accessed on 1 July 2024) [36], and SwissADME (http://www.swissadme.ch/, accessed on 1 July 2024) [37], each providing highly accurate and validated predictions. The analysis demonstrated that all molecules under investigation exhibited promising solubility, both in water (log S ranging from −3.33 to −4.26 mol/L) and in terms of Caco-2 cell permeability (log Papp between 1.25 and 0.96 cm/s × 10) (see Table S1). These findings are crucial because water solubility directly impacts drug absorption, and permeability through Caco-2 cells serves as a model for predicting intestinal absorption. Moreover, the intrinsic water solubility (log S_0_) values, which are pH-independent and critical for absorption, ranged from −4.53 to −3.54, indicating that these molecules maintain favorable solubility in their uncharged states. Further analysis revealed additional advantageous pharmacokinetic characteristics, including high intestinal absorption (~81–92% in humans) and favorable skin permeability (log Kp~−2.78–2.99). The ability of the molecules to cross the blood–brain barrier (log BB ranging from 0.02 to 0.99) and permeate the central nervous system (log PS~−2.39–2.82) underscores their potential for targeting tissues beyond peripheral organs. The clearance rates, ranging from 0.30 to 0.61 mL/min/kg, suggest efficient elimination from the body, reducing the risk of accumulation and related toxicity concerns.

Toxicological assessments further confirmed the safety profiles of the compounds. The oral rat acute toxicity (LD_50_) ranged between 2.45 and 2.88 mol/kg, while chronic toxicity (LOAEL) was observed between 0.86 and 1.52 mg/kg/day. The estimated maximum tolerated dose in humans was between −0.08 and 0.28 mg/kg/day, reflecting a wide safety margin. Additionally, these compounds exhibited non-hepatotoxic and non-toxic profiles, indicating a reduced risk of adverse effects. Collectively, these findings demonstrate that the molecules possess a favorable ADMET profile, which is vital for exhibiting drug-like properties. Building on these insights, we further scrutinized the pharmacokinetic characteristics of the molecules against Lipinski’s Rule of Five, a widely accepted benchmark for evaluating drug-likeness in early-stage drug discovery (Table 1). Lipinski’s rule highlights the significance of key physicochemical parameters—such as hydrogen bond acceptors and donors, lipophilicity (log P), and molecular weight—in predicting oral bioavailability. Adherence to these guidelines is essential for identifying compounds with a higher probability of successful oral delivery, thus optimizing the drug development process.

While Lipinski’s Rule of Five is not an infallible predictor of success of a drug, it serves as a critical framework for prioritizing molecules with the optimal characteristics for oral administration [47]. Compounds that do not meet these criteria often face substantial challenges in terms of bioavailability and pharmacokinetics during later stages of development. In this study, all the investigated molecules adhered to Lipinski’s rule, further reinforcing their drug-like properties. These molecules not only exhibited favorable solubility, permeability, and ADMET profiles but also aligned with the physicochemical constraints essential for oral bioavailability. This strong correlation with pharmacokinetic and physicochemical parameters underscores their potential as promising candidates for further investigation and development.

#### 3.2.2. Bioavailability

Building upon our pharmacokinetic evaluation, we employed the SwissADME bioavailability radar tool to gain deeper insights into the drug-like potential of our molecules. Notably, T3 emerged as a candidate with particularly favorable physicochemical properties, indicative of enhanced oral bioavailability. The bioavailability radar plot for T3 illustrated that key parameters fell within the optimal “pink area”, which signifies the desirable range for six critical physicochemical properties: flexibility, lipophilicity, solubility, polarity, molecular size, and saturation (see Supporting Information Figure S1). These parameters are pivotal in determining the suitability of a compound for oral administration [48]. For a molecule to be classified as drug-like, its radar plot must fully align with this pink area, indicating that each physicochemical property meets the preferred criteria. Specifically, the properties include a topological polar surface area (TPSA) ranging from 20 to 130 Å^2^, solubility (log S) not exceeding −6, molecular weight (MW) between 150 and 500 g/mol, a maximum of nine rotatable bonds (to indicate flexibility), a fraction of carbons in sp^3^ hybridization (saturation) of at least 0.25, and a lipophilicity (XLOGP3) value between −0.7 and +5.0. Molecules that fulfill these criteria are more likely to exhibit enhanced oral bioavailability, a crucial factor in successful drug development.

**Figure 2:**
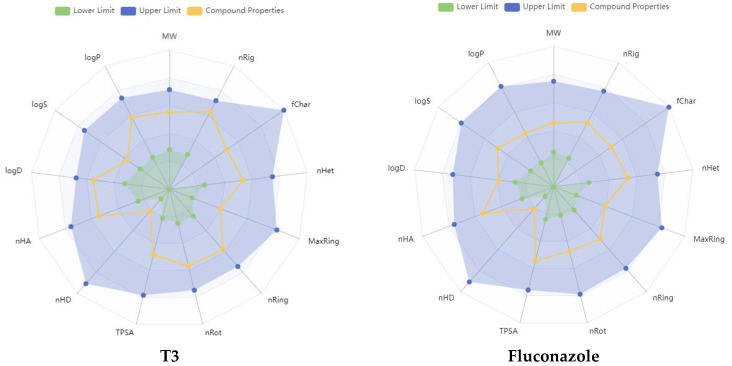
Bioavailability radar plot of the most active compound T3 and the reference drug fluconazole obtained using ADMETLab 3.0. Green represents the lower limit, while as blue represents the upper limit. The compound properties are depicted in orange.

To further substantiate these findings, we conducted a rigorous analysis of the physicochemical properties of all compounds, including T3 and the reference antifungal drug Fluconazole, using ADMETLab 3.0. This extensive evaluation encompassed 21 physicochemical properties, 19 medicinal chemistry parameters, 34 ADME endpoints, 36 toxicity endpoints, and 8 toxicophore rules. The results confirmed that all derivatives, possess optimal characteristics essential for drug-like behavior and efficient bioavailability (see Supporting Information, Figure S2). Also, as depicted in Figure 2, the parameters for T3 and the reference drug fall well within the desired ranges, reinforcing their potential as viable drug candidates. Notably, the alignment of the physicochemical profile of T3 with established criteria not only enhances its viability for further development but also highlights its promise in addressing therapeutic needs. The favorable predictions from both the SwissADME bioavailability radar and ADMETLab assessments underscore the importance of meticulous evaluation of physicochemical properties in the drug discovery process, as they form the backbone of effective pharmacokinetics and safety profiles.

#### 3.2.3. Toxicity Analysis and Safety Profiling

The toxicity profiles of the most potent derivative, T3, and the reference drug, Fluconazole, were comprehensively evaluated using the ProTox-3.0 web tool [38]. Toxicity classifications followed the globally harmonized system for chemical labeling and classification. Both compounds were categorized as Class 4, with a calculated lethal dose (LD_50_) of >500 mg/kg, confirming their non-toxic to low-toxic nature (LD_50_ values ranging from 500 to 1271 mg/kg). This classification indicates a relatively low risk of acute toxicity, suggesting minimal harm even at higher doses (see Supporting Information Figure S3). ProTox-II further provided detailed toxicity assessments for hepatotoxicity, cytotoxicity, carcinogenicity, mutagenicity, immunotoxicity, nuclear receptor signaling pathways, and stress response pathways (see Supporting Information Figure S5 and Table S2). Strikingly, both compounds exhibited a high probability of being inactive across most toxicological endpoints, though notable exceptions emerged. The derivative T3 demonstrated a potential risk of neurotoxicity, while Fluconazole exhibited a broader toxicity profile, showing predictions for hepatotoxicity, mutagenicity, and neurotoxicity, with probabilities ranging from 0.59% to 0.91%. Additionally, T3 showed a 69% probability of influencing mitochondrial membrane potential (MMP), a critical parameter in cellular function, whereas Fluconazole did not. However, Fluconazole demonstrated a significant 92.1% probability of interacting with the Aryl hydrocarbon Receptor (AhR), which could indicate potential long-term toxicological concerns.

**Figure 3:**
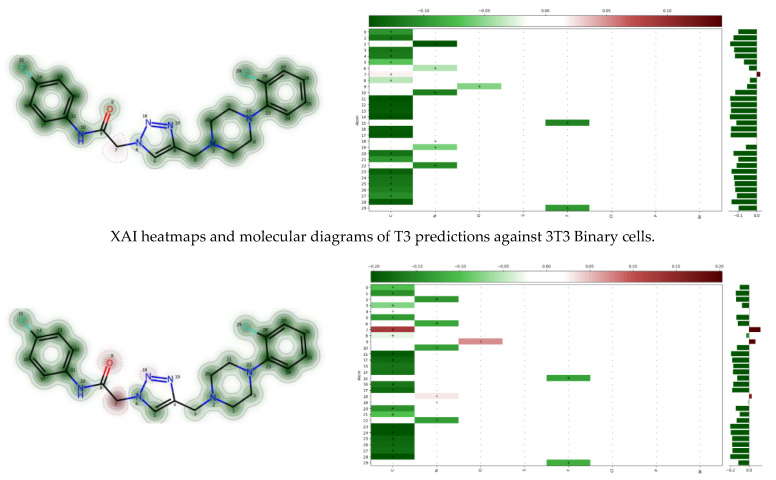

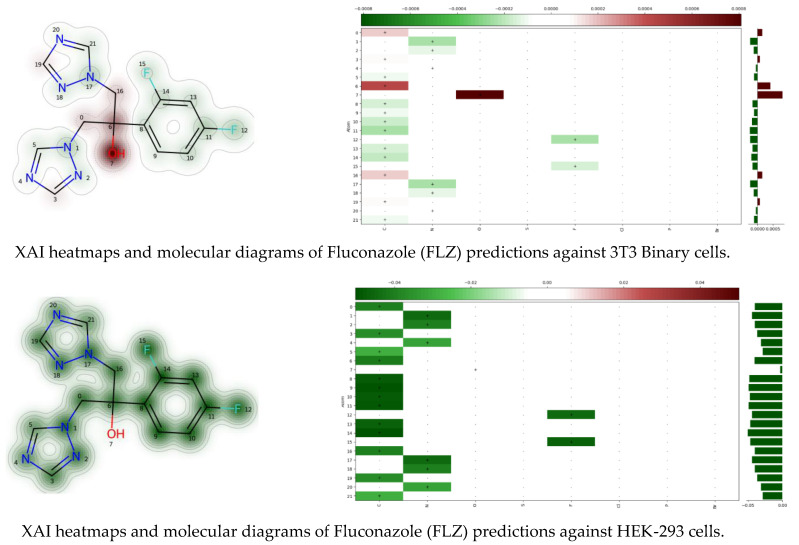
XAI heatmaps and molecular diagrams of T3 and Fluconazole (FLZ), highlighting key structural features that contribute to their classification as non-toxic compounds. The visualizations depict the influence of individual atoms on the overall toxicity profile, with green indicating toxicity-reducing features and red representing toxicity-enhancing features. Images were obtained using the cytosafe tool from LabMol.

To enhance the robustness of the toxicity predictions, BeeToxAI and CytoSafe tools were employed to provide additional safety insights. BeeToxAI, a machine-learning-based QSAR tool, predicted the acute toxicity of these compounds in honeybees, while CytoSafe focused on cytotoxicity, identifying toxicophores, which are the key structural features responsible for toxicity (see Supporting Information Figure S4). The results indicated that T3 is non-toxic with a probability of 78–85%, while fluconazole showed a higher non-toxicity prediction, ranging from 94 to 99% (Figure 3). One of the most impactful aspects of the CytoSafe analysis was the use of explainable AI (XAI) heatmaps and molecular diagrams. This advanced visualization technique offered a scientifically rigorous, yet intuitive, way to understand the contributions of individual atoms and their interactions to cytotoxicity across 3T3 Binary and HEK-293 cell lines. By leveraging Morgan Fingerprints, the XAI Molecular Diagram quantified the influence of each atom, mapped on a gradient from green (toxicity-reducing) to red (toxicity-enhancing), revealing key toxicophores within the molecular structures (see Figure 3). This dual visualization approach not only identified toxicity drivers but also provided a molecular context, allowing for targeted optimization of the derivative’s structure to mitigate potential toxic effects. These results, supported by multiple advanced toxicity prediction tools, provide a comprehensive understanding of the safety profiles of T3 and Fluconazole. The insights from XAI and molecular fingerprinting underscore the importance of structural features in driving toxicity, which could guide further refinement of T3 for enhanced therapeutic safety.

### 3.3. Biology

#### 3.3.1. Candidicidal and Bactericidal Activity of the Compounds T1–T12

The compounds T1–T12 demonstrated strong inhibitory activity against the free microbial cells of *C. auris* and *S. aureus*, both in single and dual-species culture environments. The MIC and MFC values are provided in Table 2.

Out of twelve tested compounds T3 at a very low concentration was active against individual pathogens and additionally at the same concentration T3 impacted the survival of pathogens under mixed condition. However, when compared to the single—species the mixed culture displayed higher MIC values against the investigated drugs and the present finding agree with a previous study [49].

Yeast and bacteria are often co-isolated in healthcare settings, with more than 20% of bloodstream infections caused by *C. albicans* in immunocompromised patients being polymicrobial, with *S. epidermidis* and *S. aureus* as the most frequently isolated pathogens [50]. Although the interactions between *C. auris* and other bacterial pathogens remain understudied, both are well known for their ability to form biofilms, both inside the host and on abiotic medical devices. Therefore, assessing the growth and survival of these pathogens in a polymicrobial environment is critical for developing effective treatment strategies to combat dual-species infections. In this study, we investigated the effect of T3 on the planktonic growth and biofilm formation of *C. auris* and *S. aureus* under single and dual-species conditions.

#### 3.3.2. T3 Prohibited Biofilm Formation and Smashed Mature Biofilm Formed by Single and Dual—Species

**Figure 4:**
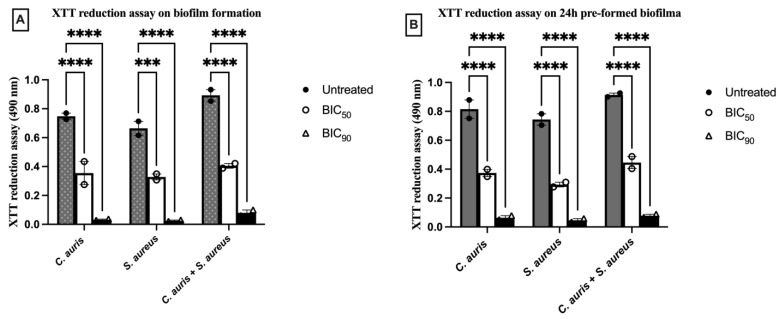
XTT reduction assay to evaluate biofilm inhibitory potential of T3 under single and dual—species condition. The absorbance was measured at 490 nm. Effect on biofilm formation (**A**) and on 24 h preformed biofilms (**B**) of individual pathogens and in combination was studies in untreated (control) and BIC_50_ and BIC_90_ of **T3**. Data were expressed as mean ± SEM and were analyzed for statistical significance using two-way ANOVA followed by Tukey’s multiple comparisons test. Significance levels are denoted by *** *p* < 0.001; **** *p* < 0.0001.

The biofilm inhibitory effect of T3 was assessed by measuring the reduced metabolic activity of cells embedded in single and dual-species biofilms, as compared to the control, using the XTT reduction assay (Figure 4). Notably, a significant reduction in biofilm formation by *C. auris* and *S. aureus*, both individually and in combination, was observed. Moreover, T3 exhibited a strong impact on disrupting 24 h preformed biofilms when compared to the control. The inhibitory effect of T3 on biofilm formation and preformed biofilms varied between single- and dual-species biofilms. The results indicated that the 24 h mature biofilm formed by *C. auris* and *S. aureus* showed the highest tolerance to T3; however, the compound was still effective in eradicating the 24 h mature biofilm compared to the control (Table 3).

The collaboration between yeast and bacteria results in elevated antimicrobial tolerance level [13,15,51] and therefore, the polymicrobial infections are associated with higher mortality rates contrary to their mono-microbial condition [13]. Although, the interaction between *C. auris* and other microbes are not explored in detail, but the ability of *C. albicans* and *S. aureus* to form biofilms both individually as well as in mixed conditions has been well documented. Previous research has highlighted similar interactions between *Candida* and *Staphylococcus* species [52,53,54], prompting us to explore the in vitro interactions between *C. auris* and *S. aureus* under biofilm-forming conditions. Additionally, we assessed the anti-biofilm activity of T3 against these pathogens in both single-species and mixed-species environments. It is well-established that the collaboration between yeast and bacteria in biofilms is synergistic, enhancing each other’s growth and promoting the upregulation of various pathogenic traits, including drug resistance [52]. Targeting the interkingdom interactions and disrupting biofilm formation could provide an effective strategy to mitigate the issues associated with mixed biofilms. Our results indicated that T3, at its half MIC (19.53 µg/mL), effectively inhibited biofilm formation, while at 4 × MIC (156.25 µg/mL), it was successful in eradicating mature biofilms formed by the dual-pathogen species. This characteristic offers T3 a significant advantage over conventional antimicrobials, which often have a limited spectrum of activity and are commonly linked to the development of drug resistance.

#### 3.3.3. T3 Impact over Cellular Viability of Pathogens in Single and Dual—Species Biofilm

**Figure 5:**
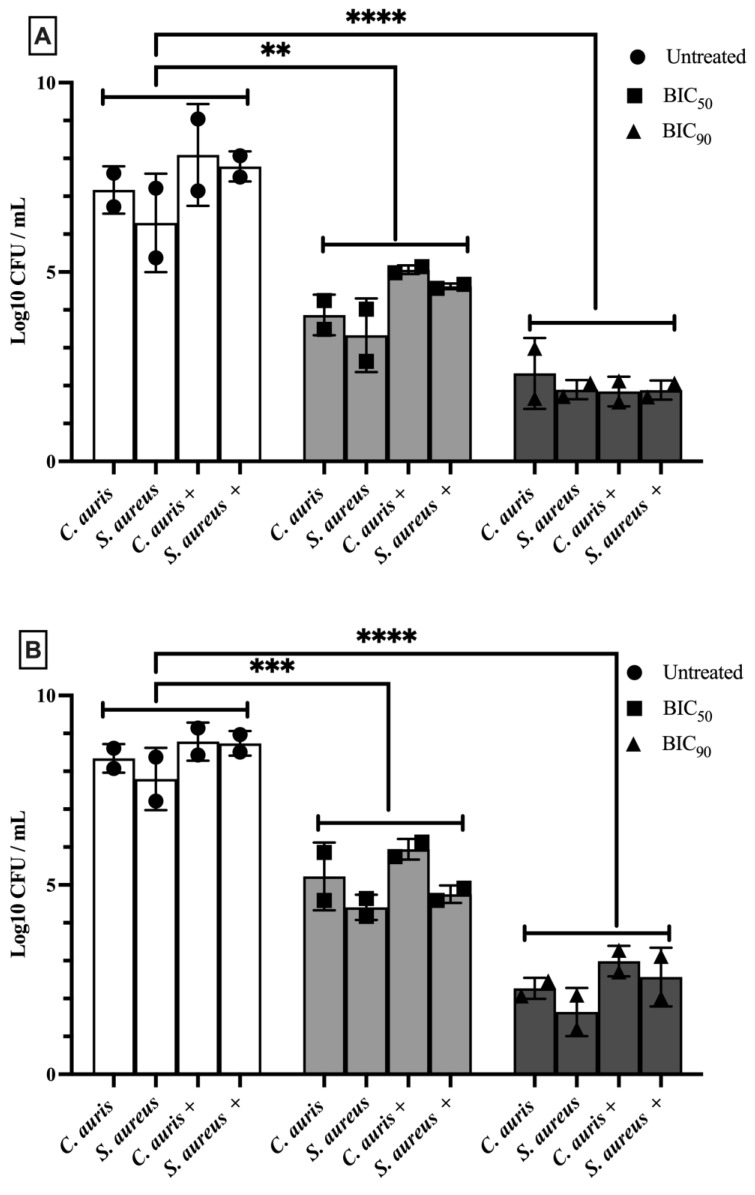
Viability assay of single and dual—species biofilms at different concentrations of T3. Cells embedded in the dual—species biofilm of *C. auris* and *S. aureus* (*C. auris* +, viable cells of *C. auris* in mixed biofilms; *S. aureus* +, viable cells of *S. aureus* in mixed biofilms). (**A**) Cellular viability during biofilm formation. (**B**) Cellular viability in 24 h preformed biofilm. Data were expressed as mean ± SEM and were analyzed for statistical significance using two-way ANOVA followed by Dunnett’s multiple comparisons test. Significance levels are denoted by ** *p* < 0.01, *** *p* < 0.001; **** *p* < 0.0001.

Although XTT method is considered a gold standard assay and being widely used by researchers for detecting the antibiofilm potency of antimicrobial compounds [55,56], in present investigation, we also considered log CFU/mL as an additional parameter to authenticate the viability of yeast and bacterial cells from single and dual—species biofilms. Moreover, log CFU counts helps in establishing a quantitative foundation for the presence of viable cell in the biofilms and have been quite regularly used in studies related to mixed biofilms [57,58]. According to the data recorded, the log CFU/mL agreed with the XTT assay and also suggested that T3 is definitely having anti-biofilm property, and its activity is directly related on the dislodging of biofilms and not killing the cells present in the biofilms. T3 exhibited a concentration-dependent inhibition of both biofilm formation and preformed biofilms in single and dual-species cultures, with a significant reduction in log CFU counts observed as the concentration of the compound increased (Figure 5). To further investigate the mode of antimicrobial action of T3 on the biofilm structure, we performed CLSM and SEM analyses.

#### 3.3.4. T3 Drastically Effects Total Biomass in Single and Dual—Species Biofilm

**Figure 6:**
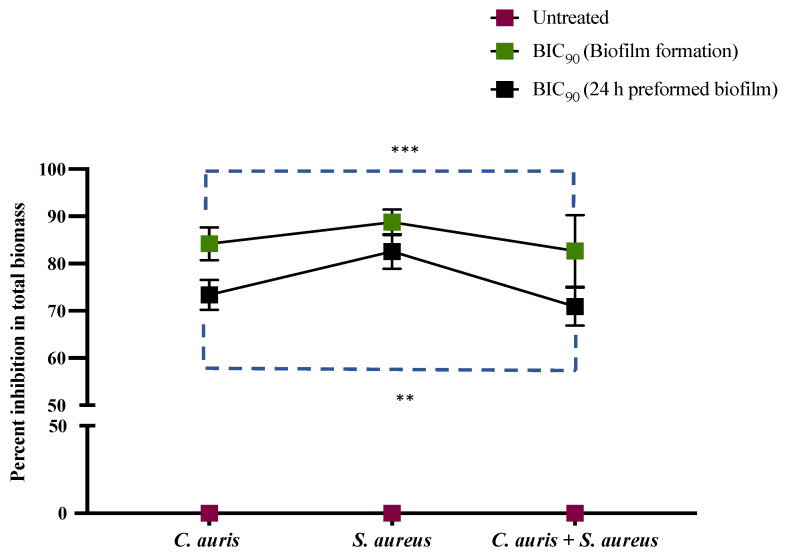
Percent inhibition in total biomass of formation and 24 h preformed dual—species biofilm of *C. auris* and *S. aureus*. Violet crystal (VC) staining method was adopted to evaluate the biofilm disruptive property of T3 at various concentrations. Percent inhibition in total biomass in untreated control was considered 0%.**, *p* < 0.005; ***, *p* < 0.0005.

**Figure 7:**
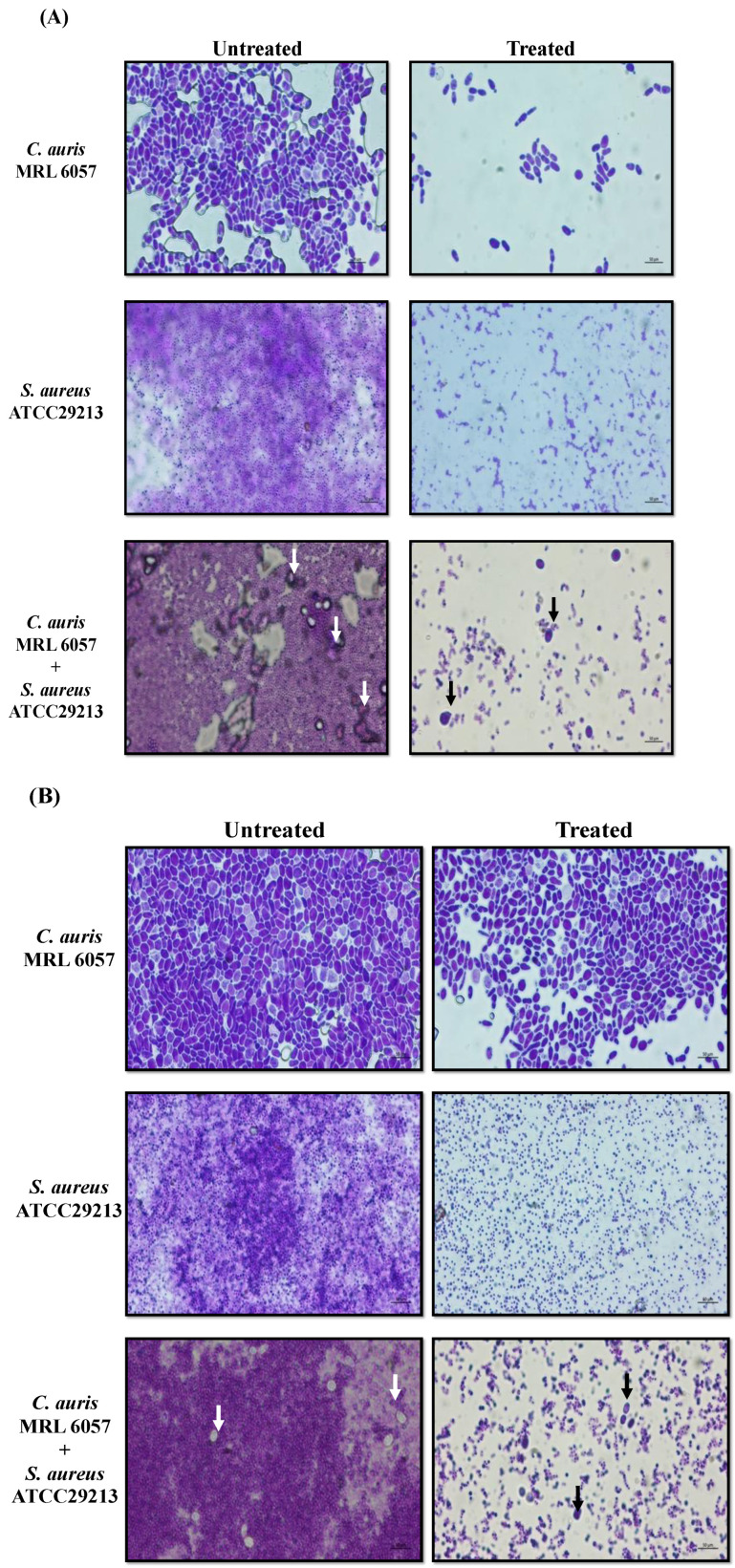
Light micrograph of biofilm formed by *C. auris* and *S. aureus* under monomicrobial and mixed condition. The figure displays, (**A**) the inhibitory effect of T3 on biofilm formation and (**B**) effect on 24 h preformed biofilm. White and black arrows denote the presence of *C. auris* in the dual—species biofilm. Scale bar = 50 µm.

T3 significantly lowered the amount of total biomass formed in both single and mixed culture biofilms (Figure 6). The obtained results advocated that T3 not only inhibited the growth and viability of biofilm under mixed condition it also impaired the 24 h preformed biofilm attached to the substratum and therefore, created an adverse environment for the propagation of *C. auris* and *S. aureus* both individually and in mixed environment. However, the 24 h biofilm was found to be more tolerant to the test compound as compared to the biofilm in the forming stage. Moreover, the data captured further clarified that there was around 82% and 71% inhibition in total biomass of 24 h mature biofilm formed by the dual—species suggesting the raising tolerance of mature biofilm compared to the newly formed biofilms. The anti-biofilm activity of T3 against both single and dual-species biofilms further highlights its potential as an effective anti-biofilm agent. Its ability to target polymicrobial biofilms may reduce the likelihood of resistance development in *C. auris* and *S. aureus*, particularly when compared to commonly used antibiotics. Additionally, light microscopy images of the biofilms revealed a significant reduction in biofilm biomass, with very few cells remaining adhered to the coverslip (Figure 7). This characteristic of T3 is particularly valuable in clinical settings, as biofilm-associated infections caused by *C. auris* and *S. aureus* are notoriously difficult to treat.

#### 3.3.5. Con-A and FUN-1 Staining Further Confirmed the Anti-Biofilms Property of T3

**Figure 8:**
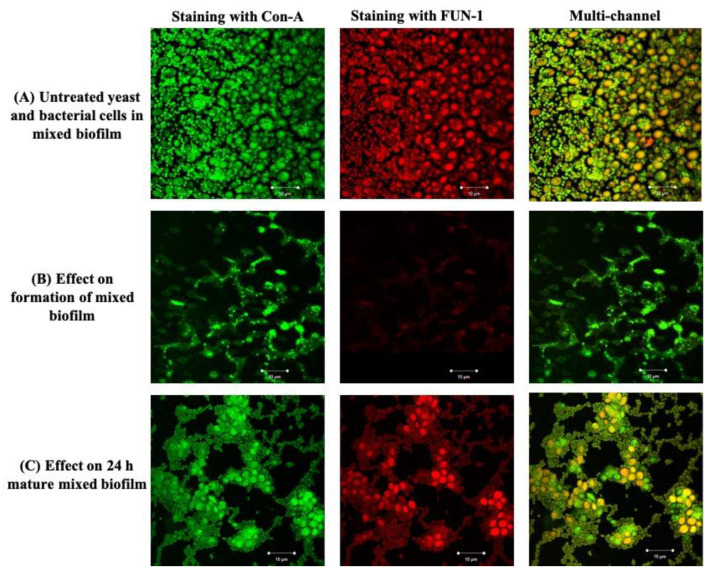
CLSM analysis of dual—species biofilm stained with Con-A and FUN-1. The figure represents the anti-biofilm potential of T3 against biofilm formation, and 24 h mature polymicrobial biofilms formed by *C. auris* and *S. aureus*. The images show yeast and bacterial cells embedded in the dual–species biofilms that is captured at 63× oil immersion objective at ×2.0 magnification. Scale bar = 10 µm.

To further validate the ability of T3 to inhibit biofilm formation and disrupt 24 h mature biofilms formed by C. auris and S. aureus, we stained the biofilms with a combination of fluorescent dyes: FUN-1 (a cytoplasmic probe for assessing cell viability) and Con-A (which specifically binds to cell wall polysaccharides). These staining agents have been previously used to examine the viability of cells within biofilms [41]. Bright green fluorescence indicated the incorporation of Con-A into the cell wall polysaccharides in the dual-species biofilms, while red fluorescence revealed the integration of FUN-1 in the metabolically active yeast and bacterial cells. Concisely, the sections of the slide fluorescing orange-red in the multi-channel mode represented the presence of metabolically active bacterial and yeast cells, contrary to this, the section showed bright green fluorescence depicts the presence of biofilm matrix and those sections that were yellow-green in color represented the presence of dead cells (as FUN-1 remained confined to the cytosol) [41]. Herein the results are represented as untreated dual—species biofilm control (Figure 8A), exposure of T3 during biofilm formation (Figure 8B) and exposure of T3 on 24 h mature biofilm (Figure 8C). The micrographs obtained for untreated dual—specie biofilms were having dense and compact biofilm architecture which when observed under multichannel mode can gave a bright red-green fluorescence throughout the slide. In contrast, the treated samples exhibited a significantly different outcome. In the presence of the BIC90 concentration of T3, there was a noticeable reduction in both biofilm density and live cell counts, with more yellow-green fluorescence observed, indicating an increase in non-viable cells (Figure 8B,C). Furthermore, the damage was more pronounced in the biofilm formation samples compared to the 24 h mature biofilms formed under dual-species conditions. In the treated biofilm formation samples, cellular size was reduced, clear depressions were observed, and the faded red fluorescence further confirmed the non-viability of both yeast and bacterial cells in the dual-species biofilms. These findings support our previous results, reinforcing the anti-biofilm efficacy of T3 against the critical dual-species biofilms.

#### 3.3.6. SEM for Structural Analysis of Dual—Species Biofilms upon Treatment with T3

**Figure 9:**
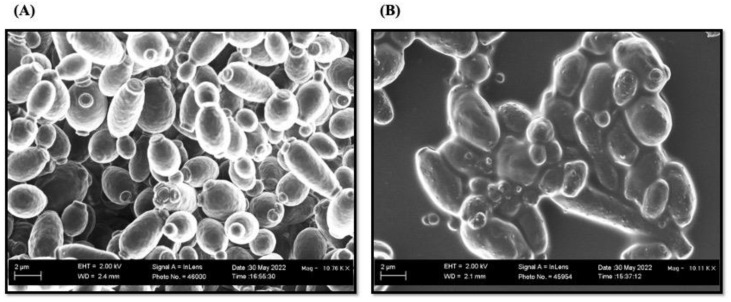
SEM micrographs dual-species biofilms formed by *C. auris* and *S. aureus*. (**A**) Untreated mixed biofilms; (**B**) treated with T3 at BIC_90_.

The three—dimensional structure of cells embedded in dual—specie biofilms were observed both in untreated and treated conditions (Figure 9). The untreated 24 h preformed biofilms were intact, dense and had a complex network of yeast and bacterial cells making it impenetrable (Figure 9A). On the other hand, the view of treated 24 h mature biofilms were solely different, distorted and wrinkled bacterial and yeast cells were observed at BIC_90_ of T3 (Figure 9B).

SEM analysis provided valuable insights into the effect of T3 on the morphology of cells in dual-species biofilms, highlighting significant differences between treated and untreated biofilms. Previous studies have shown a synergistic interaction between *C. albicans* and *S. aureus* in polymicrobial biofilms [59], which aligns with the observed behavior of *C. auris* and *S. aureus* in mixed biofilm conditions. In our study, untreated dual-species biofilms displayed a substantial amount of extracellular matrix, which is commonly associated with increased antimicrobial resistance in polymicrobial cultures. Similar findings have been reported in recent research, where enhanced biofilm matrix production in mixed biofilms contributed to elevated drug resistance [60]. In contrast, treatment with T3 disrupted cell morphology, reduced the biofilm matrix, and allowed better penetration of the compound into the bacterial and yeast cells embedded in the biofilm, leading to the eradication of the mixed biofilm.

#### 3.3.7. Cytotoxicity

**Figure 10:**
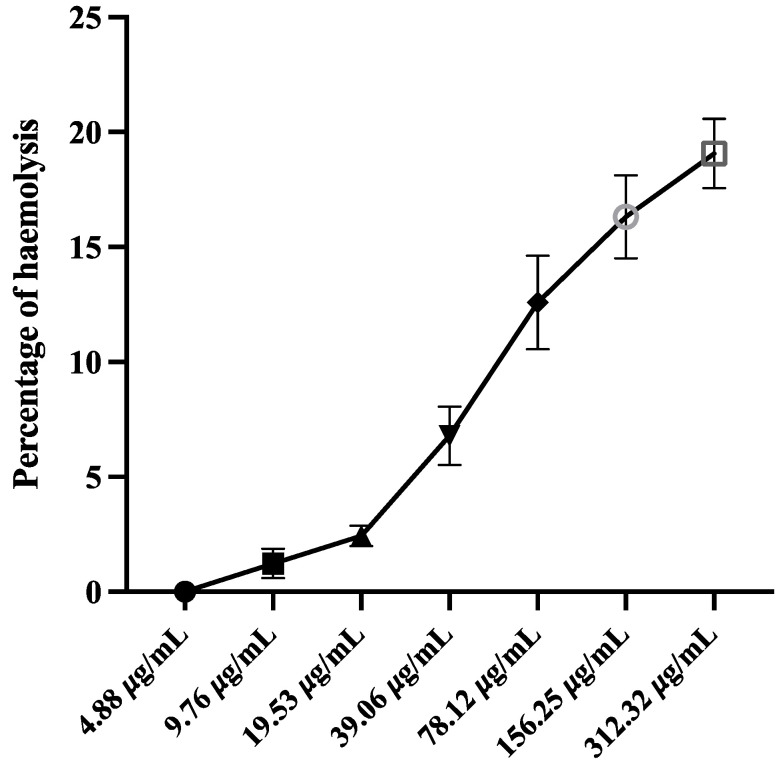
Hemolytic activity of T3. Hemolysis of RBCs by T3 at various concentrations. *p* < 0.0001.

Hemolysis is a critical test to assess the safety profile of drugs. To evaluate the safety of T3, its potential to induce hemolysis was tested using horse red blood cells (RBC). For comparison, PBS and Triton X-100 were used as negative and positive controls, representing 0% and 100% hemolysis, respectively. T3 at a concentration of 4.88 μg/mL showed no hemolytic activity against erythrocytes. At higher concentrations, hemolysis ranged from 1.6% to 19.07% (Figure 10). These results demonstrate the low cytotoxicity of T3, supporting its potential for use in in vivo studies.

#### 3.3.8. Cytotoxicity of T3 Against RAW 264.7 Cells

In addition to the demonstrated potent antimicrobial and antibiofilm properties of T3 in previous studies, it was essential to establish its cytotoxicity profile to ensure its safety for subsequent in vivo evaluations. The MTT assay, a widely used and reliable method for assessing cell viability and cytotoxicity, was employed to evaluate the impact of varying concentrations of T3 on mammalian RAW 264.7 macrophage cells.

**Figure 11:**
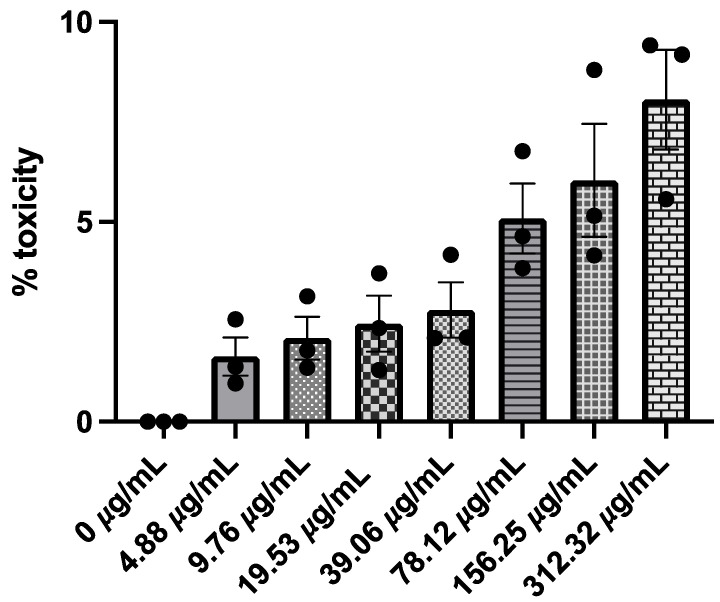
Evaluation of the cytotoxicity of T3 against RAW 264.7 cells using the MTT assay. The cytotoxicity analysis of various concentrations of T3 against RAW 264.7 cells.

The results revealed that T3 exhibited a minimal cytotoxic effect on RAW 264.7 cells, with a dose-dependent response observed (Figure 11). Specifically, at the highest tested concentration of 312.32 µg/mL, the average cytotoxicity was measured at 8.06%, indicating that over 90% of the cell population remained viable under these conditions (Figure 11). These findings suggest that T3 exerts a shallow level of cytotoxicity, even at relatively high concentrations, which is a critical consideration for its potential therapeutic application.

The low cytotoxicity observed aligns with the requirements for compounds intended for in vivo applications, as it ensures minimal off-target effects on mammalian cells. Based on these results, T3 meets the preliminary safety criteria and is a strong candidate for advancing to the next stage of evaluation in in vivo models.

## 4. Conclusions

*Candida auris* is a frequently isolated yeast pathogen found on the skin of both immunocompetent and immunocompromised individuals, and it has the capacity to interact with bacterial species, such as *Staphylococcus aureus*, in the same ecological niche. Our study confirms this interaction, leading to the formation of robust dual-species biofilms. Among the tested compounds, T3 emerged as the most effective against planktonic cells of both *C. auris* and *S. aureus*, in both single and dual-species culture conditions. T3 significantly disrupted biofilm formation and reduced microbial viability within these biofilms. Notably, the physicochemical analysis of T3 revealed favorable bioavailability and permeability properties, which further highlight its therapeutic potential. This study is one of the few that explores anti-biofilm efficacy against both single and dual-species biofilms of *C. auris* and *S. aureus*, setting the stage for future research aimed at developing alternative therapeutic strategies to tackle polymicrobial infections. Further studies testing additional strains will strengthen the conclusion and claims of this finding.

## Figures and Tables

**Table 1:** Lipinski and Veber filters for drug-like characteristics.

Compound	M.Wt	Clog P *	Log D ^#^	HBA	HBD	RBs	tPSA	Ro5 (Y/N)
T1	394.45	2.378	2.38	5	1	7	66.29	Y
T2	408.47	2.510	2.74	5	1	7	66.29	Y
T3	412.44	2.517	2.29	6	1	7	66.29	Y
T4	428.89	3.489	3.34	5	1	7	66.29	Y
T5	428.89	3.031	2.82	5	1	7	66.29	Y
T6	473.34	3.140	2.95	5	1	7	66.29	Y
T7	462.18	3.396	3.19	7	1	8	66.29	Y
T8	462.44	3.396	3.17	8	1	8	66.29	Y
T9	424.47	2.386	2.48	6	1	8	75.52	Y
T10	439.44	2.286	2.46	7	1	8	112.11	Y
T11	439.44	2.286	2.33	7	1	8	112.11	Y
T12	463.34	3.684	3.19	5	1	7	66.29	Y
^†^ Fluconazole	306.27	0.50	0.56	7	1	5	81.93	Y

* At pH 7.4; **^#^** Determined with ChemAxonLogD predictor; ^†^ standard drug; M.Wt: Molecular Weight; HBA: hydrogen bond acceptor; HBD: hydrogen bond donor, obtained by Marvin Sketch 23.16; RB: Rotatable bonds; Ro5 (Y/N): Rule of five followed or not; Y: Yes; N: No; Veber filter: Rotatable bonds ≤ 10, tPSA ≤ 140 in Å^2^.

**Table 2:** Antimicrobial susceptibility profiling of the test compounds (T1–T12).

Test	*C. auris* MRL6057		*S. aureus* ATCC29213		*C. auris* MRL6057 + *S. aureus* ATCC29213	
	Median MIC (µg/mL)	Median MFC (µg/mL)	Median MIC (µg/mL)	Median MBC (µg/mL)	Median MIC (µg/mL)	Median MFC/MBC (µg/mL)
T1	156.25	625.0	78.12	156.25	1250	2500
T2	312.5	625.0	156.25	312.5	1250	2500
T3	9.76	19.53	2.44	2.44	78.12	78.12
T4	78.12	78.12	78.12	78.12	625.0	625.0
T5	19.53	39.06	19.53	19.53	156.25	312.5
T6	39.06	78.12	39.06	78.12	312.5	312.5
T7	156.25	312.5	156.25	312.5	312.5	625.0
T8	78.12	312.5	39.06	312.5	625.0	625.0
T9	312.5	625.0	156.25	312.5	625.0	1200
T10	78.12	312.5	19.53	39.06	156.25	625.0
T11	78.12	156.25	156.25	312.5	312.5	625.0
T12	312.5	1250	312.5	625	1250	2500
AmB	4.0	8.0	NA	NA	NT	NT
AZI	NT	NT	0.5	0.5	NT	NT

Each value represents the median of three independent experiments. AmB: Amphotericin B; AZI: Azithromycin; NT: Not tested.

**Table 3:** Biofilm inhibitory concentrations (BIC) of T3 against single and dual—species biofilm (during their formation and 24 h preformed) of *C. auris* and *S. aureus*.

T3	Biofilm Formation			24 h Preformed Biofilm		
	*C. auris*	*S. aureus*	*C. auris* + *S. aureus*	*C. auris*	*S. aureus*	*C. auris* + *S. aureus*
BIC_50_	9.76 μg/mL (0.25× MIC)	4.88 μg/mL (0.25× MIC)	9.76 μg/mL (0.25× MIC)	39.06 μg/mL (MIC)	9.76 μg/mL (0.5× MIC)	78.12 μg/mL (2× MIC)
BIC_90_	19.53 μg/mL (0.5× MIC)	9.76 μg/mL (0.5× MIC)	19.53 μg/mL (0.5× MIC)	78.12 μg/mL (2× MIC)	39.06 μg/mL (2× MIC)	156.25 μg/mL (4× MIC)

## Data Availability

The original contributions presented in the study are included in the article/Supplementary Material, further inquiries can be directed to the corresponding authors.

## Supplementary Materials

The following supporting information can be downloaded at: https://www.mdpi.com/article/10.3390/pharmaceutics16121570/s1, Figure S1: The bioavailability radar plot for the derivatives T1–T12 illustrating key parameters within the optimal “pink area”, which signifies the desirable range for six critical physicochemical properties: flexibility, lipophilicity, solubility, polarity, molecular size, and saturation; Figure S2: Bioavailability radar plots of all the derivatives obtained using ADMETLab 3.0 web tool. Green represents the lower limit, while as blue represents the upper limit. The compound properties are depicted in orange; Figure S3: Oral toxicity prediction results and comparison of T3 and Fluconazole using ProTox-II tool; Figure S4: Predicted acute toxicity of the most active compound T3 and the reference drug Fluconazole in honeybees using BeeToxAI, a machine-learning-based QSAR tool; Figure S5: Toxicity profile analysis of the most active compound T3 and the reference drug using the toxicity model computation tool and online database; Table S1: ADMET [Absorption, distribution, metabolism, excretion, and toxicity] Parameters of the compounds; Table S2: Toxicity profile analysis of the most active compounds using the toxicity model computation tool and online database.
