# Supplementary material for: Concomitant Inhibition and Collaring of Dual-Species Biofilms Formed by Candida auris and Staphylococcus aureus by Triazole Based Small Molecule Inhibitors
Source: Pharmaceutics. 2024 Dec 8;16(12):1570. doi: 10.3390/pharmaceutics16121570 (PMC11677466; doi:10.3390/pharmaceutics16121570)
Supplement: Supplementary file 1 [file pharmaceutics-16-01570-s001.zip › pharmaceutics-3307287-supplementary.pdf]

pharmaceutics

Article

Supplementary Materials: Concomitant Inhibition and Collaring of Dual-Species Biofilms Formed by *Candida auris* and *Staphylococcus aureus* by Triazole Based Small Molecule Inhibitors

Humaira Parveen, Sayeed Mukhtar, Mona O. Albalawi, Syed Khasim, Aijaz Ahmad and Mohmmad Younus Wani

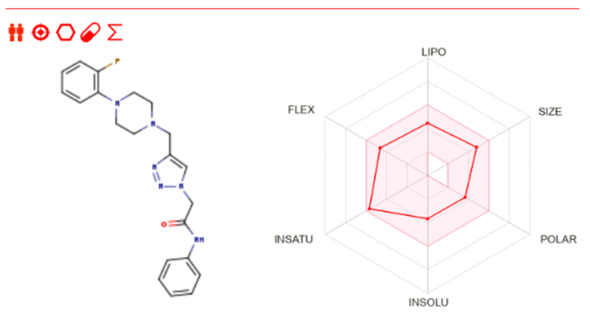	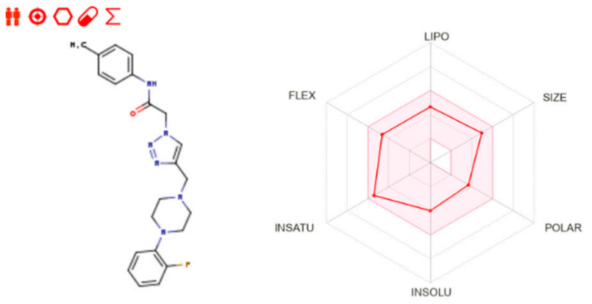
**T1**	**T2**
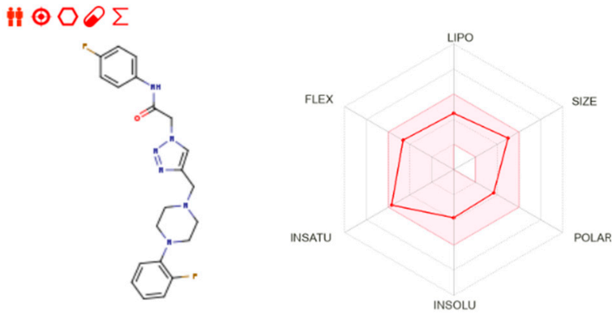	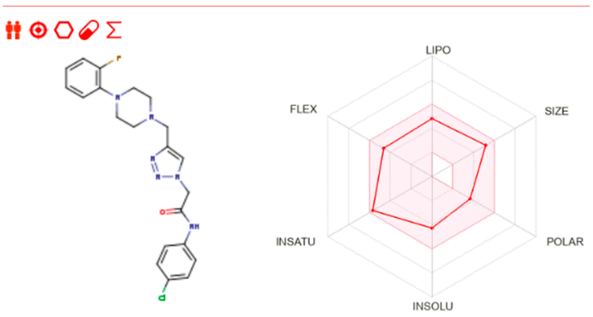
**T3**	**T4**
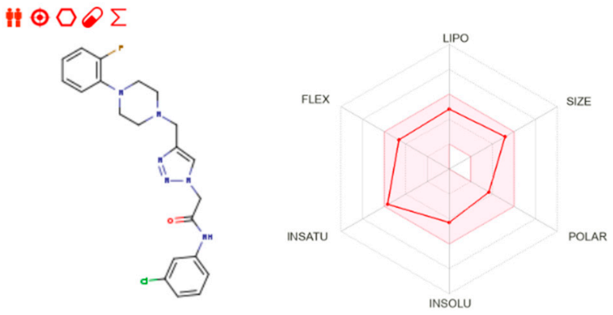	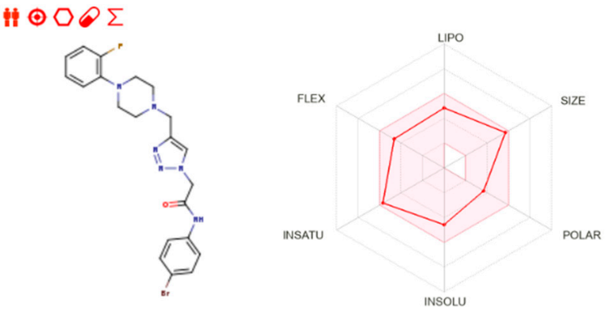
**T5**	**T6**

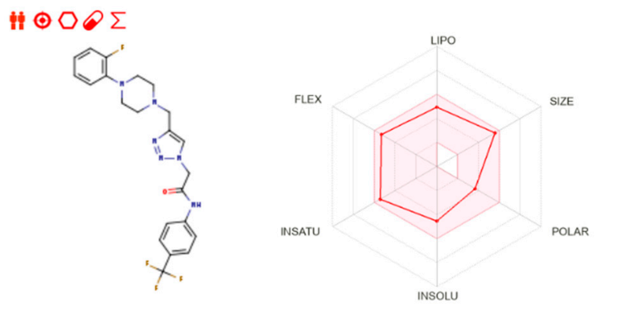

T7

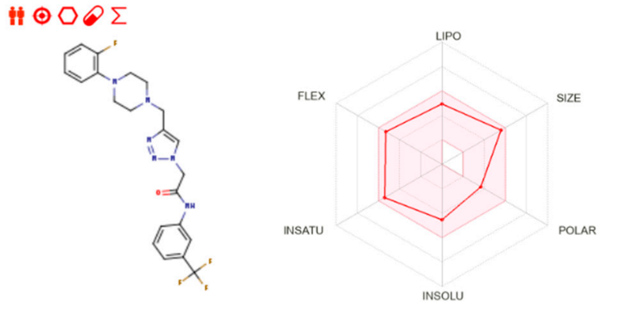

T8

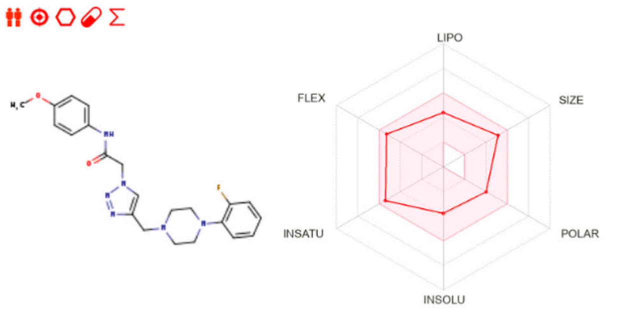

T9

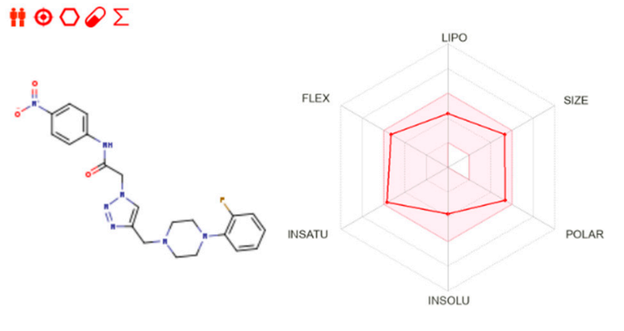

T10

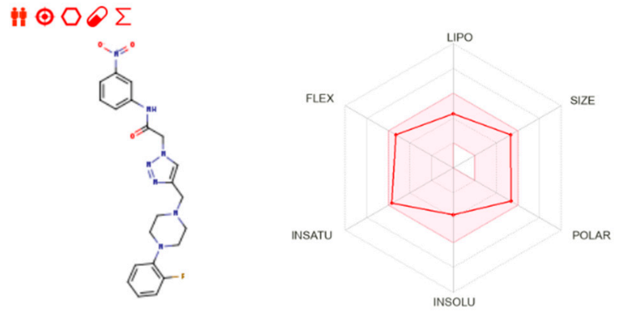

T11

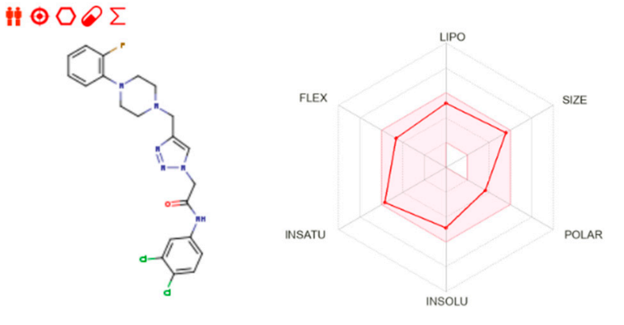

T12

Figure S1. The bioavailability radar plot for the derivatives T1-T12 illustrating key parameters within the optimal "pink area," which signifies the desirable range for six critical physicochemical properties: flexibility, lipophilicity, solubility, polarity, molecular size, and saturation.

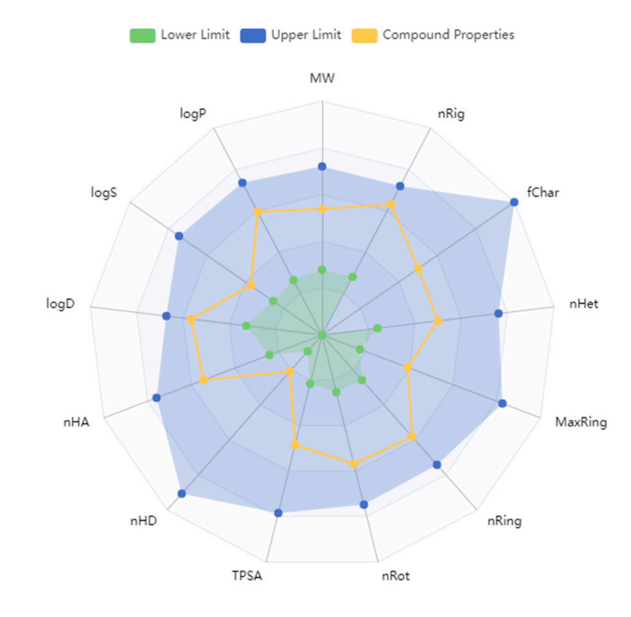
Lower Limit
Upper Limit
Compound Properties
MW
nRig
fChar
nHet
MaxRing
nRing
nRot
TPSA
nHD
nHA
logD
logS
logP

T1

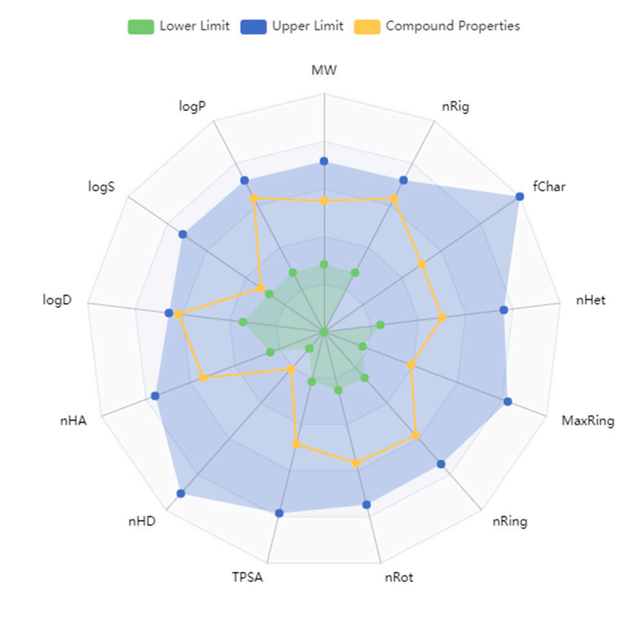
Lower Limit
Upper Limit
Compound Properties
MW
nRig
fChar
nHet
MaxRing
nRing
nRot
TPSA
nHD
nHA
logD
logS
logP

T2

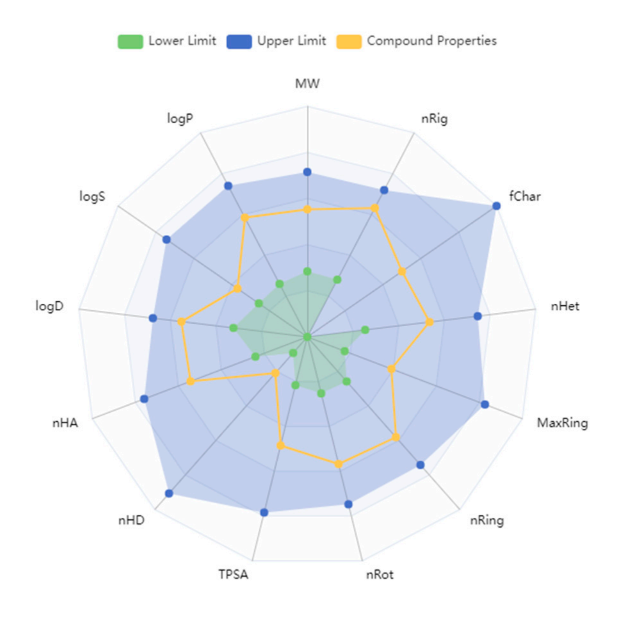
Lower Limit
Upper Limit
Compound Properties
MW
nRig
fChar
nHet
MaxRing
nRing
nRot
TPSA
nHD
nHA
logD
logS
logP

T3

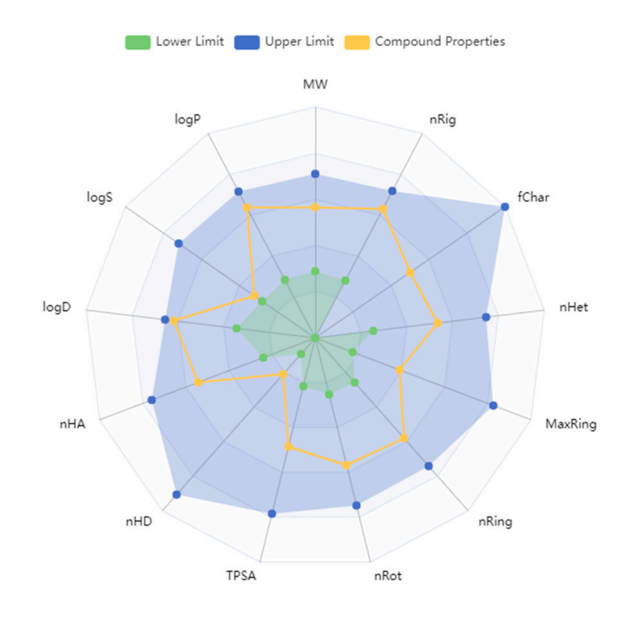
Lower Limit
Upper Limit
Compound Properties
MW
nRig
fChar
nHet
MaxRing
nRing
nRot
TPSA
nHD
nHA
logD
logS
logP

T4

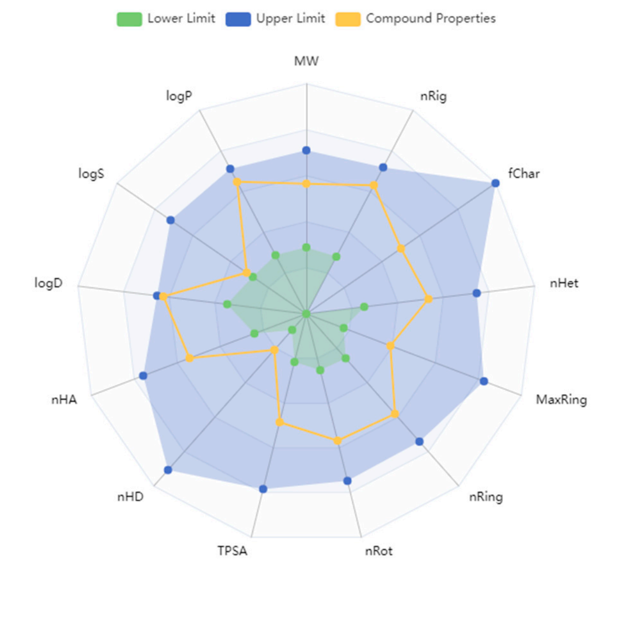
Lower Limit
Upper Limit
Compound Properties
MW
nRig
fChar
nHet
MaxRing
nRing
nRot
TPSA
nHD
nHA
logD
logS
logP

T5

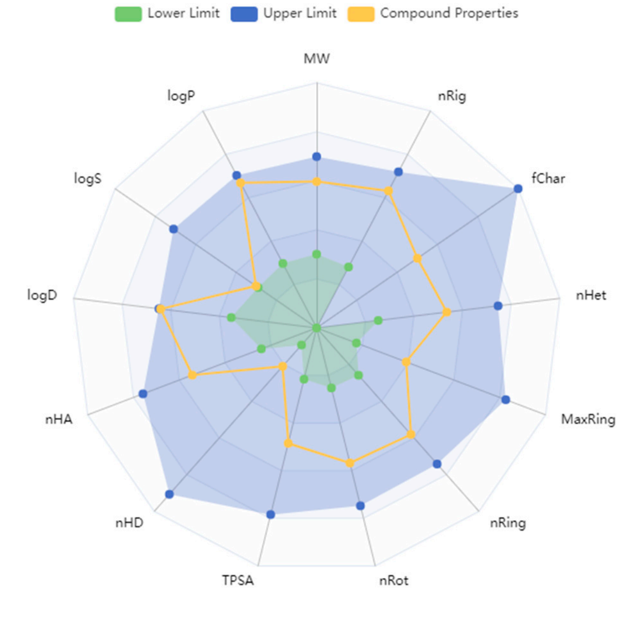
Lower Limit
Upper Limit
Compound Properties
MW
nRig
fChar
nHet
MaxRing
nRing
nRot
TPSA
nHD
nHA
logD
logS
logP

T6

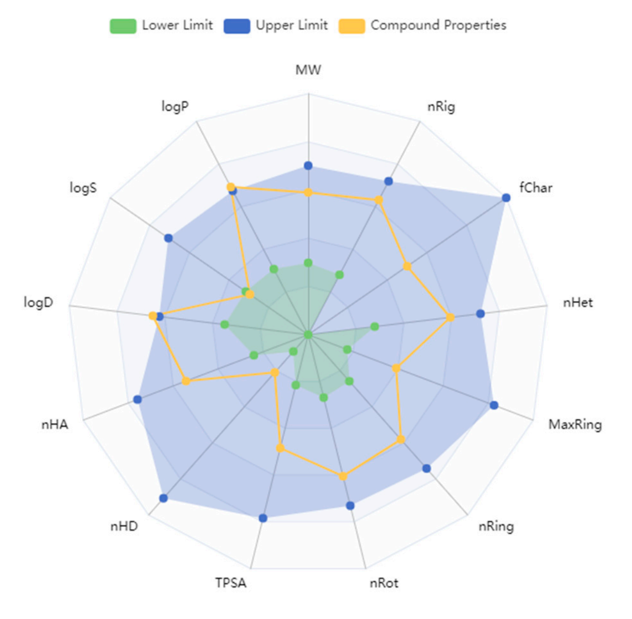
Lower Limit
Upper Limit
Compound Properties
MW
nRig
fChar
nHet
MaxRing
nRing
nRot
TPSA
nHD
nHA
logD
logS
logP

T7

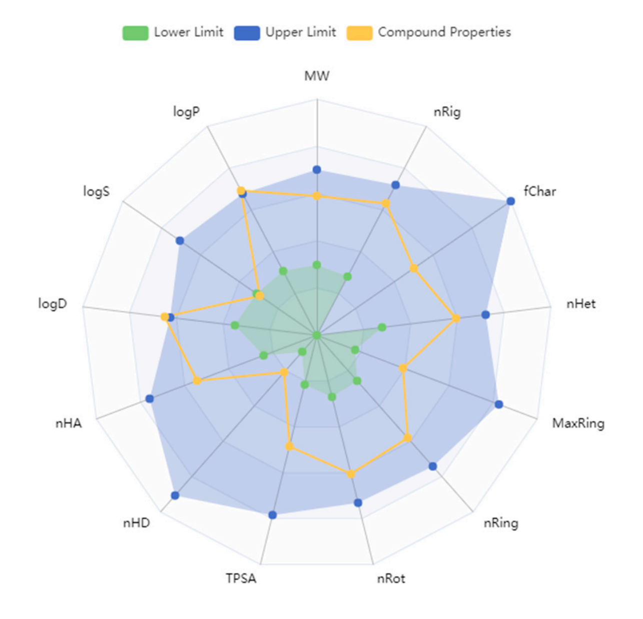
Lower Limit
Upper Limit
Compound Properties
MW
nRig
fChar
nHet
MaxRing
nRing
nRot
TPSA
nHD
nHA
logD
logS
logP

T8

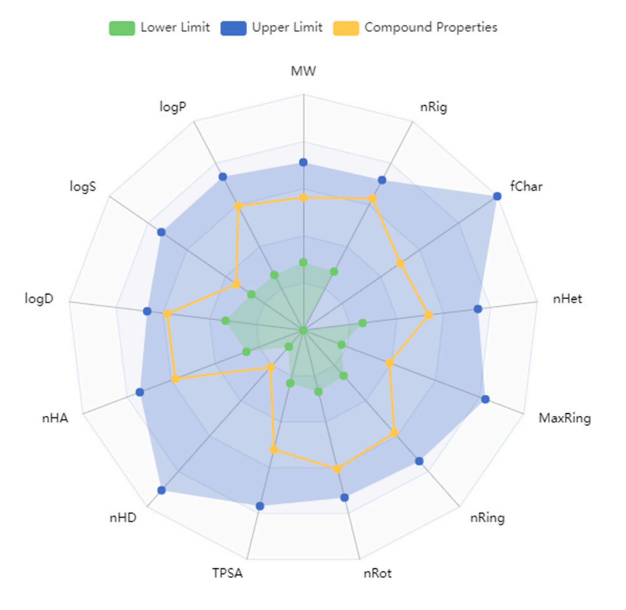

T9

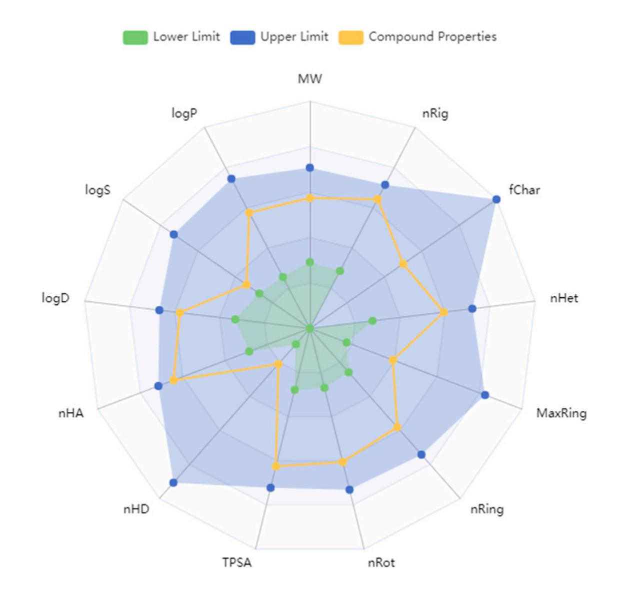

T10

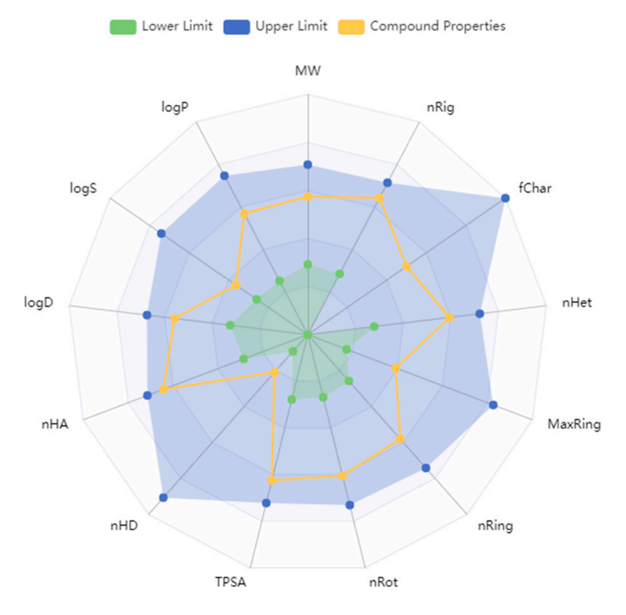

T11

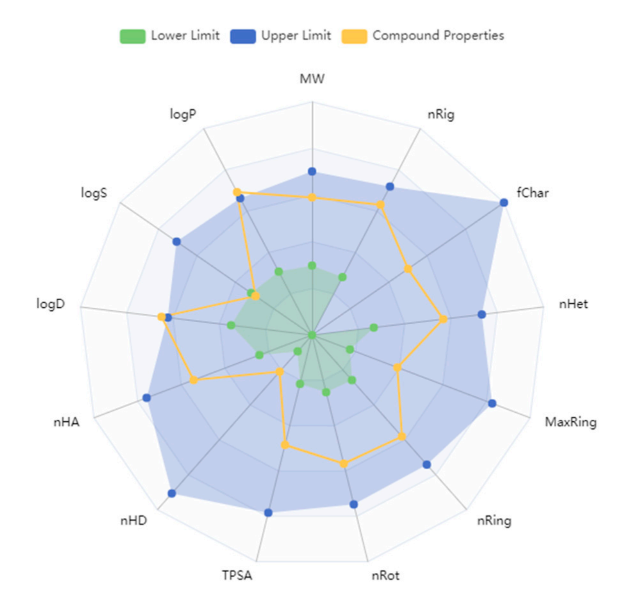

T12

Figure S2. Bioavailability radar plots of all the derivatives obtained using ADMETLab 3.0 web tool. Green represents the lower limit, while as blue represents the upper limit. The compound properties are depicted in orange.

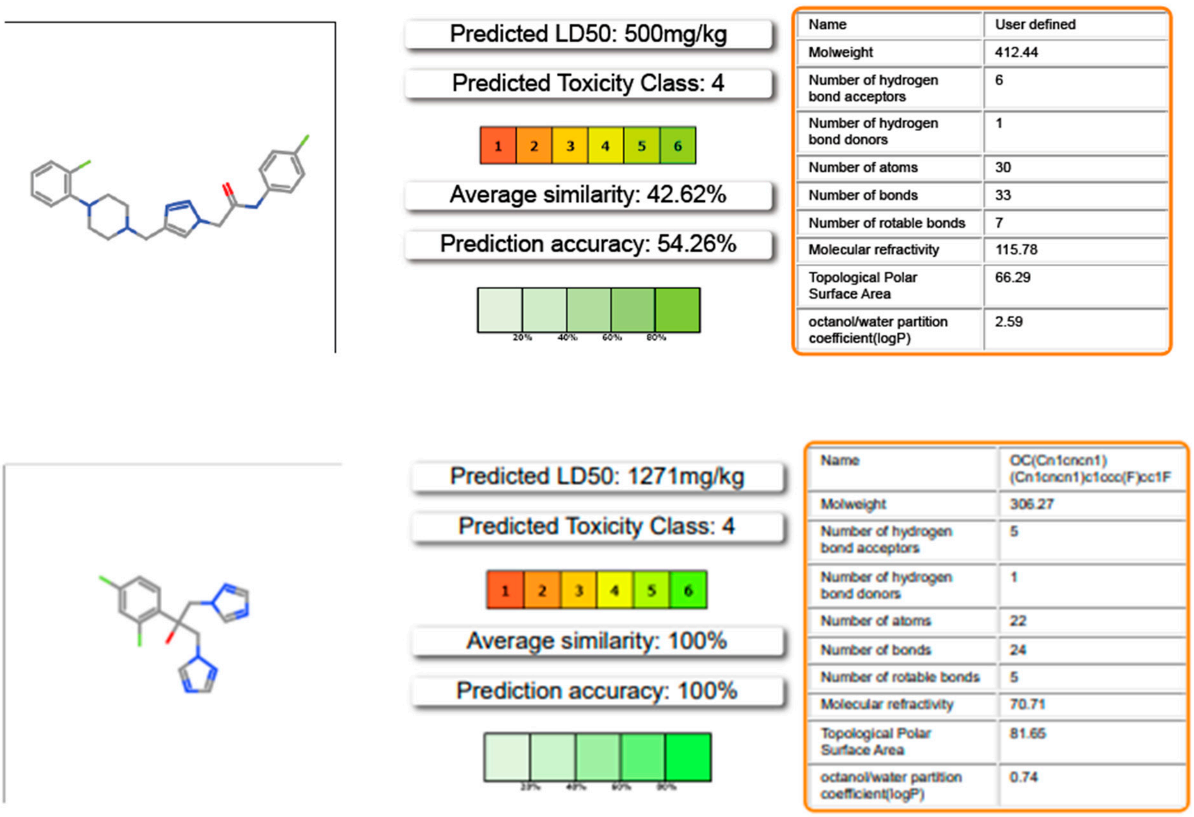

Figure S3. Oral toxicity prediction results and comparison of T3 and Fluconazole using ProTox-II tool.

Model #	Enpoint	Prediction (Confidence)	Applicability Domain	Contribution Mapping
1	Acute oral Toxicity<br>**Assay type**: Acute Oral Toxicity Test (OECD 213)<br>**Animal**: Honey bee *(Apis mellifera)*<br>***ML Algorithm****: Random forest*<br>***Descriptors****: MACCS*	**Non-toxic (-)**<br>(84.0%)	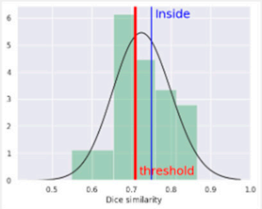	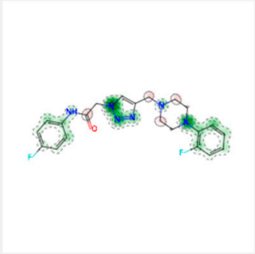
3	Acute Contact Toxicity<br>**Assay type**: Acute Contact Toxicity Test (OECD 214)<br>**Animal**: Honey bee *(Apis mellifera)*<br>***ML Algorithm****: SVM*<br>***Descriptors****: FeatMorgan FCFP2 with bit-vector size of 2048 bits with radius of 2*	**Non-toxic (-)**<br>(96.0%)	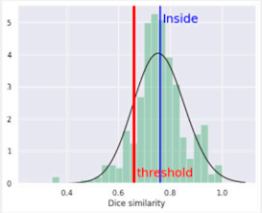	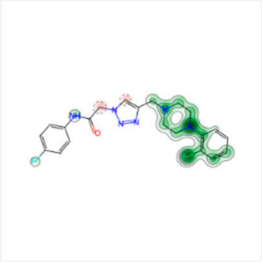

T3

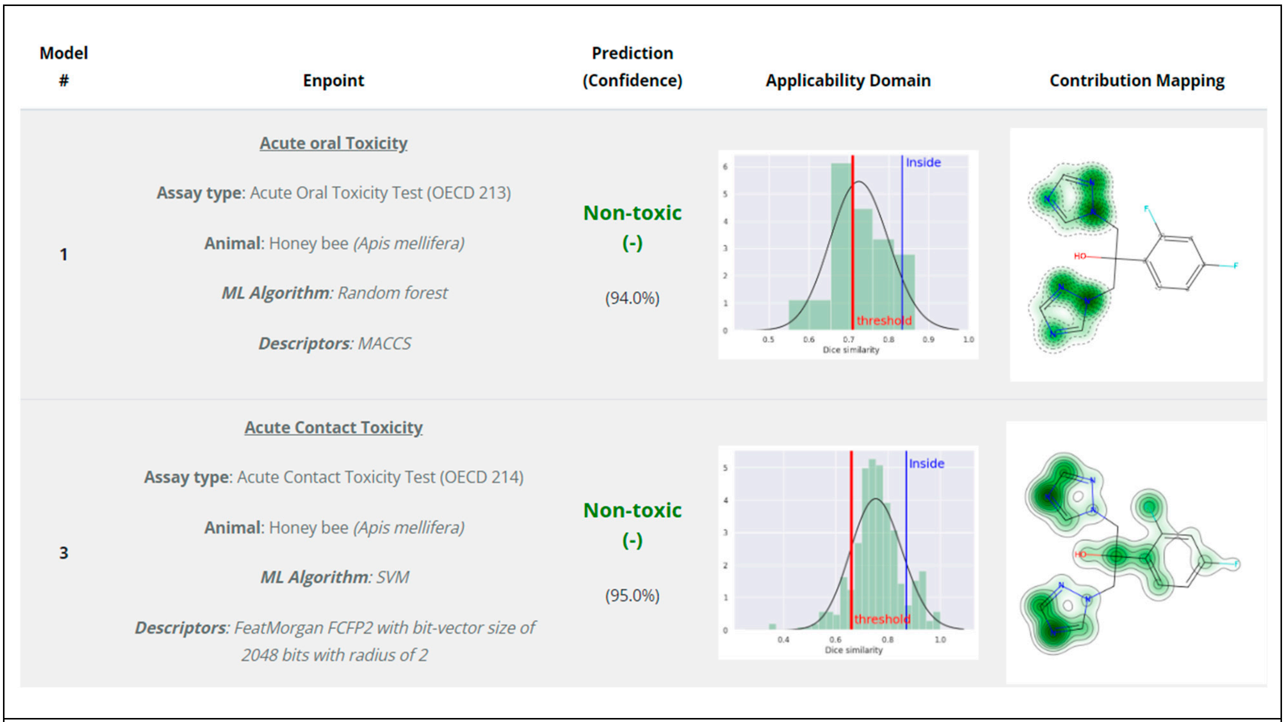

Fluconazole

Figure S4. Predicted acute toxicity of the most active compound T3 and the reference drug Fluconazole in honeybees using BeeToxAI, a machine-learning-based QSAR tool.

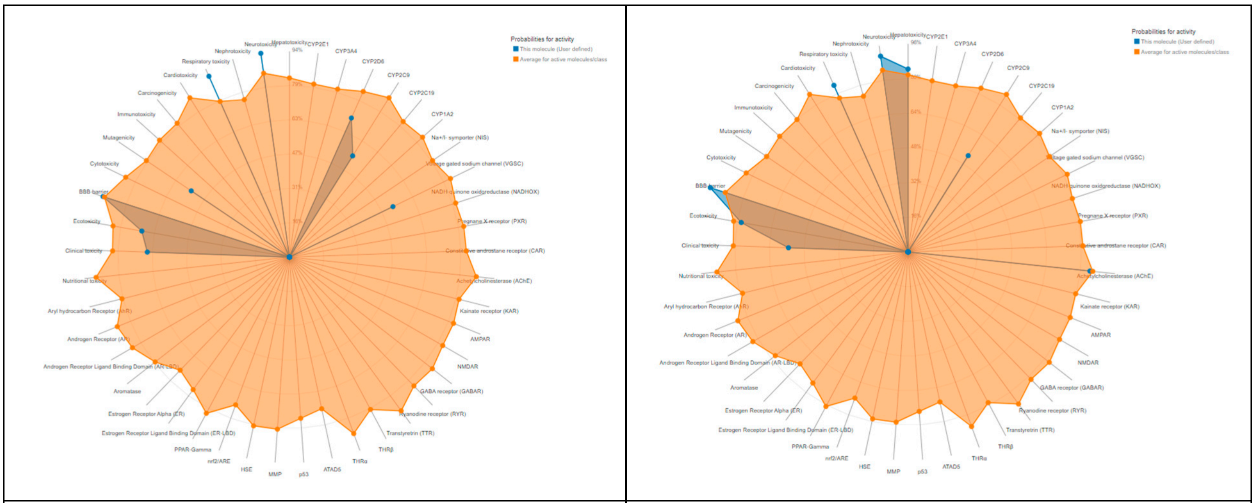

T3	Fluconazole

Figure S5. Toxicity profile analysis of the most active compound T3 and the reference drug using the toxicity model computation tool and online database.

Table S1. ADMET [Absorption, distribution, metabolism, excretion, and toxicity] Parameters of the compounds.

ADMET Parameters	T1	T2	T3	T4	T5	T6	T7	T8	T9	T10	T11	T12	FLZ*
**Absorption**													

ADMET Parameters	T1	T2	T3	T4	T5	T6	T7	T8	T9	T10	T11	T12	FLZ*
**Water Solubility**	-3.33	-3.80	-3.82	-3.81	-3.85	-3.89	-4.24	-4.26	-3.67	-4.07	-4.08	-4.37	-2.41
**$CaCO_2$ Permeability**	1.01	1.24	1.25	1.10	1.02	1.10	0.96	0.96	1.20	1.0	0.98	1.04	1.04
**Intestinal Absorption**	91.0	92.6	92.0	89.7	90.3	89.6	87.6	87.8	93.0	81.1	81.1	89.4	78.38
**Skin Permeability**	-2.92	-2.99	-2.97	-2.91	-2.91	-2.91	-2.85	-2.85	-2.86	-2.78	-2.78	-2.91	-2.76
**Distribution**													
**BBB permeability**	0.34	0.04	0.02	0.29	0. 26	0.29	0.16	0.17	-0.71	-1.00	-0.99	0.23	-1.31
**CNS permeability**	-2.55	-2.54	-2.65	-2.44	-2.44	-2.42	-2.39	-2.39	-2.81	-2.82	-2.82	-2.33	-3.18
**PPB†**	91.7	91.2	90.3	96.9	97.4	94.5	95.6	96.3	89.9	85.2	92.8	98.3	90.5
**VDss‡**	1.04	1.14	0.98	0.62	0.99	1.35	1.66	1.29	1.01	1.27	0.95	1.06	0.79
**Metabolism**													
**CYP2D6 substrate**	Yes	No	No	No	No	No	No	No	No	No	No	No	No
**CYP2C9 inhibitor**	No	No	No	No	No	No	No	No	No	Yes	No	No	No
**CYP2D6 inhibitor**	No	No	No	Yes	Yes	Yes	Yes	Yes	No	No	No	Yes	No
**Excretion**													
**Total Clearance**	0.59	0.34	0.54	0.58	0.51	1.66	0.43	0.39	0.61	0.35	0.30	0.60	0.34
**CL_{plasma}**	4.06	3.53	3.37	3.39	3.24	2.37	5.02	5.17	4.68	3.01	3.84	3.38	6.01
**‡$T_{1/2}$**	0.56	0.55	0.68	0.66	0.64	0.82	0.54	0.52	0.44	0.72	0.59	0.66	0.81
**Toxicity**													
**Oral Rat Acute Toxicity (LD_{50})**	2.61	2.51	2.45	2.69	2.76	2.70	2.77	2.78	2.44	2.88	2.88	2.84	2.20
**Oral Rat Chronic Toxicity (LOAEL)**	1.30	0.86	0.99	1.22	1.23	1.21	1.30	1.29	0.90	1.52	1.52	1.16	0.92
**Max. tolerated dose (human)**	-0.19	-0.14	-0.08	-0.24	-0.24	-0.24	-0.31	-0.32	0.28	-0.11	-0.11	-0.26	0.12
**Hepatotoxicity**	Yes	Yes	Yes	Yes	Yes	Yes	Yes	Yes	Yes	Yes	Yes	Yes	No
**AMES toxicity**	No	No	No	No	No	No	No	No	No	No	No	No	No

*FLZ: Fluconazole was the standard drug used in the studies.

†PPB: Plasma Protein Binding; Optimal: < 90%, Drugs with high protein-bound may have a low therapeutic index.

‡VDss: Volume Distribution; Optimal: 0.04-20L/kg

CLplasma: The unit of predicted CLplasma penetration is mL/min/kg, >15 mL/min/kg: High clearance, 5-15 mL/min/kg: Moderate clearance, <5 mL/min/kg: Low clearance.

‡ The unit of predicted half-life is hours (h), ultra short half-life drugs: ½ to < 1h; short half-life drugs: 1 to 4h; intermediate short half-life drugs: 4 to 8h; long half-life drugs: >8h.

Table S2: Toxicity profile analysis of the most active compounds using the toxicity model computation tool and online database.

		T3		FLZ	
**Classification**	**Target**	Prediction	Probability	Prediction	Probability
**Organ Toxicity**	Hepatotoxicity	Inactive	0.54	Active	0.84
**Toxicity end points**	Carcinogenicity	Inactive	0.53	Inactive	0.62
	Immunotoxicity	Inactive	0.96	Inactive	0.83
	Mutagenicity	Inactive	0.53	Active	0.59
	Cytotoxicity	Inactive	0.75	Inactive	0.75
	Neurotoxicity	Active	0.94	Active	0.91
**Tox21-Nuclear receptor signaling pathways**	Aryl hydrocarbon Receptor (AhR)	Inactive	0.87	Active	0.92
	Androgen Receptor (AR)	Inactive	0.98	Inactive	0.98
	Androgen Receptor Ligand Binding Domain (AR-LBD)	Inactive	0.96	Inactive	0.99
	Aromatase	Inactive	0.80	Active	0.72
	Estrogen Receptor Alpha (ER)	Inactive	0.81	Inactive	0.92
	Estrogen Receptor Ligand Binding Domain (ER-LBD)	Inactive	0.96	Inactive	0.99
	Peroxisome Proliferator Activated Receptor Gamma (PPAR-Gamma)	Inactive	0.94	Inactive	0.99
**Tox21-Stress response pathways**	Nuclear factor (erythroid-derived 2)-like 2/antioxidant responsive element (nrf2/ARE)	Inactive	0.95	Inactive	0.97
	Heat shock factor response element (HSE)	Inactive	0.95	Inactive	0.97
	Mitochondrial Membrane Potential (MMP)	Active	0.69	Inactive	0.92
	Phosphoprotein (Tumor Supressor) p53	Inactive	0.91	Inactive	0.97
	ATPase family AAA domain-containing protein 5 (ATAD5)	Inactive	0.97	Inactive	0.99

Characterization data of the derivatives 3 and T1-T12

2-(4-((4-(2-fluorophenyl)piperazin-1-yl)methyl)-1H-1,2,3-triazol-1-yl)acetyl chloride **(3)**

Yield: 93%; Anal. Calc. For $C_{15}H_{17}ClFN_5O$: C 53.34, H 5.07, N 20.73%; found: C 53.45, H 5.12, N 20.75%; FTIR $\nu_{max}cm^{-1}$: 3185 (C-H triazole ring), 3087 (CH, Ar), 1725 (C=O), 1560 (C=C, Ar), 1342, 1315 (C-N), 1120, 1050 (C-C); ^{1}H NMR (DMSO-d_6) δ(ppm): 7.76 (1H, s, triazole ring), 7.36-7.22 (m, 2H, Ph-Pz ring), 6.98-6.94 (m, 2H, Ph-Pz ring), 5.05 (2H, s, CH_2,), 4.01 (2H, s, CH_2), 3.38-3.07 (m, 8H, Piperazine ring); ^{13}CNMR (DMSO-d_6) δ(ppm): 168.0 (C=O), 157.5, 155.6, 143.8 (C-triazole), 141.4, 141.3, 137.6, 129.0, 124.4, 123.9, 123.3 (C-triazole), 120.1, 118.5, 116.0, 115.8, 52.9, 52.7, 50.3, 49.5; ESI-MS m/z: [M^++H] 338.11.

***2-(4-((4-(2-fluorophenyl)piperazin-1-yl)methyl)-1H-1,2,3-triazol-1-yl)-N-phenylacetamide* (T1)**

Yield: 87%; Anal. Calc. For $C_{21}H_{23}FN_6O$: C 63.94, H 5.88, N 21.31%; found: C 64.04, H 5.85, N 21.40%; FTIR $\nu_{max}cm^{-1}$: 3180 (C-H triazole ring), 3082 (CH, Ar), 1725 (C=O), 1565 (C=C, Ar), 1348, 1310 (C-N), 1120, 1049 (C-C); ^{1}H NMR (DMSO-*d*$_6$) δ(ppm): 8.96 (s, 1H, NH), 7.77 (1H, s, triazole ring), 7.53-7.42 (m, 5H, Ph), 7.36-7.12 (m, 4H, Ph-Pz ring), 5.23 (2H, s, CH_2,), 4.01 (2H, s, CH_2), 3.34-3.12 (m, 8H, Piperazine ring); ^{13}CNMR (DMSO-*d*$_6$) δ(ppm): 167.8 (C=O), 157.5, 155.6, 143.7 (C-triazole), 141.3, 124.4, 124.2, 123.3 (C-triazole), 118.5, 116.0, 120.1, 118.5, 116.0, 115.8, 57.3, 52.7, 50.3, 49.4; ESI-MS m/z: [M^++H] 395.19.

***2-(4-((4-(2-fluorophenyl)piperazin-1-yl)methyl)-1H-1,2,3-triazol-1-yl)-N-(p-tolyl)acetamide* (T2)**

Yield: 88%; Anal. Calc. For $C_{22}H_{25}FN_6O$: C 64.69, H 6.17, N 20.57%; found: C 64.69, H 6.22, N 20.60%; FTIR $\nu_{max}cm^{-1}$: 3185 (C-H triazole ring), 3082 (CH, Ar), 1720 (C=O), 1565 (C=C, Ar), 1340, 1312 (C-N), 1123, 1049 (C-C); ^{1}H NMR (DMSO-*d*$_6$) δ(ppm): 8.96 (s, 1H, NH), 7.78 (1H, s, triazole ring), 7.53-7.42 (m, 4H, Ph), 7.36-7.12 (m, 4H, Ph-Pz ring), 5.23 (2H, s, CH_2,), 4.01 (2H, s, CH_2), 3.34-3.07 (m, 8H, Piperazine ring), 2.35 (3H, s, CH_3); ^{13}CNMR (DMSO-*d*$_6$) δ(ppm): 168.1 (C=O), 157.5, 155.6, 143.8 (C-triazole), 141.4, 136.6, 132.4, 129.9, 124.4, 124.1, 123.9, 123.3 (C-triazole), 120.1, 118.5, 116.0, 115.8, 52.9, 50.3, 49.5, 20.7; ESI-MS m/z: [M^++H] 409.21.

***N-(4-fluorophenyl)-2-(4-((4-(2-fluorophenyl)piperazin-1-yl)methyl)-1H-1,2,3-triazol-1-yl)acetamide* (T3)**

Yield: 75%; Anal. Calc. For $C_{21}H_{22}F_2N_6O$: C 61.16, H 5.38, N 20.38%; found: C 61.25, H 5.40, N 20.40%; FTIR $\nu_{max}cm^{-1}$: 3185 (C-H triazole ring), 3080 (CH, Ar), 1725 (C=O), 1558 (C=C, Ar), 1340, 1315 (C-N), 1118, 1050 (C-C); ^{1}H NMR (DMSO-*d*$_6$) δ(ppm): 9.67 (s, 1H, NH), 7.77 (1H, s, triazole ring), 7.52-7.42 (m, 4H, Ph), 7.36-7.12 (m, 4H, Ph-Pz ring), 5.23 (2H, s, CH_2,), 4.01 (2H, s, CH_2), 3.34-3.07 (m, 8H, Piperazine ring); ^{13}CNMR (DMSO-*d*$_6$) δ(ppm): 168.0 (C=O), 157.5, 155.6, 143.8 (C-triazole), 141.4, 141.3, 137.6, 129.0, 124.4, 123.9, 123.3 (C-triazole), 120.1, 118.5, 116.0, 115.8, 52.9, 52.7, 50.3, 49.5; ESI-MS m/z: [M^++H] 413.18.

***N-(4-chlorophenyl)-2-(4-((4-(2-fluorophenyl)piperazin-1-yl)methyl)-1H-1,2,3-triazol-1-yl)acetamide* (T4)**

Yield: 85%; Anal. Calc. For $C_{21}H_{22}ClFN_6O$: C 58.81, H 5.17, N 19.59%; found: C 58.90, H 5.15, N 19.55%; FTIR $\nu_{max}cm^{-1}$: 3182 (C-H triazole ring), 3085 (CH, Ar), 1725 (C=O), 1565 (C=C, Ar), 1340, 1315 (C-N), 1125, 1049 (C-C); ^{1}H NMR (DMSO-*d*$_6$) d(ppm): 9.55 (s, 1H, NH), 7.78 (1H, s, triazole ring), 7.61-7.34 (m, 4H, Ph), 7.25-6.94 (m, 4H, Ph-Pz ring), 5.23 (2H, s, CH_2,), 4.01 (2H, s, CH_2), 3.34-3.07 (m, 8H, Piperazine ring); ^{13}CNMR (DMSO-*d*$_6$) δ(ppm): 168.0 (C=O), 157.5, 155.6, 143.8 (C-triazole), 141.4, 141.3, 137.6, 129.0, 124.4, 123.9, 123.3 (C-triazole), 120.1, 118.5, 116.0, 115.8, 52.9, 52.7, 50.3, 49.5; ESI-MS m/z: [M^++H] 429.15.

N-(3-chlorophenyl)-2-(4-((4-(2-fluorophenyl)piperazin-1-yl)methyl)-1H-1,2,3-triazol-1-yl)acetamide (T5)

Yield: 82%; Anal. Calc. For $C_{21}H_{22}ClFN_6O$: C 58.81, H 5.17, N 19.59%; found: C 58.90, H 5.20, N 19.60%; FTIR $\nu_{max}cm^{-1}$: 3185 (C-H triazole ring), 3085 (CH, Ar), 1722 (C=O), 1560 (C=C, Ar), 1340, 1310 (C-N), 1120, 1049 (C-C); ^{1}H NMR (DMSO-*d*$_6$) δ(ppm): 9.55 (s, 1H, NH), 7.76 (1H, s, triazole ring), 7.61-7.31 (m, 4H, Ph), 7.36-7.12 (m, 8H, Ph-Pz ring), 5.23 (2H, s, CH_2,), 4.01 (2H, s, CH_2), 3.34-3.07 (m, 4H, Piperazine ring); ^{13}CNMR (DMSO-*d*$_6$) δ(ppm): 168.0 (C=O), 157.5, 155.6, 143.8 (C-triazole), 141.4, 141.3, 137.6, 129.0, 124.4, 123.9, 123.3 (C-triazole), 120.1, 118.5, 116.0, 115.8, 52.9, 52.7, 50.3, 49.5; ESI-MS m/z: [M^++H] 429.15.

***N-(4-bromophenyl)-2-(4-((4-(2-fluorophenyl)piperazin-1-yl)methyl)-1H-1,2,3-triazol-1-yl)acetamide* (T6)**

Yield: 75%; Anal. Calc. For $C_{21}H_{22}BrFN_6O$: C 53.29, H 4.68, N 17.75%; found: C 53.24, H 4.65, N 17.80%; FTIR $\nu_{max}cm^{-1}$: 3180 (C-H triazole ring), 3085 (CH, Ar), 1722 (C=O), 1558 (C=C, Ar), 1340, 1310 (C-N), 1120, 1049 (C-C); ^{1}H NMR (DMSO-*d*$_6$) δ(ppm): 9.56 (s, 1H, NH), 7.78 (1H, s, triazole ring), 7.51-7.46 (m, 4H, Ph), 7.36-6.94 (m, 4H, Ph-Pz ring), 5.23 (2H, s, CH_2,), 4.01 (2H, s, CH_2), 3.34-3.07 (m, 8H, Piperazine ring); ^{13}CNMR (DMSO-*d*$_6$) δ(ppm): 168.2 (C=O), 157.5, 155.6, 143.8 (C-triazole), 141.4, 137.6, 131.8, 124.4, 124.1, 123.3 (C-triazole), 121.8, 118.8, 118.5, 116.0, 52.9, 52.7, 50.3, 49.5; ESI-MS m/z: [M^++H] 473.10.

***2-(4-((4-(2-fluorophenyl)piperazin-1-yl)methyl)-1H-1,2,3-triazol-1-yl)-N-(4-(trifluoromethyl)phenyl)acetamide* (T7)**

Yield: 80%; Anal. Calc. For $C_{22}H_{22}F_4N_6O$: C 57.14, H 4.80, N 18.17%; found: C 57.20, H 4.85, N 18.25%; FTIR $\nu_{max}cm^{-1}$: 3185 (C-H triazole ring), 3085 (CH, Ar), 1722 (C=O), 1562 (C=C, Ar), 1345, 1315 (C-N), 1120, 1048 (C-C); ^{1}H NMR (DMSO-*d*$_6$) δ(ppm): 9.57 (s, 1H, NH), 7.77 (1H, s, triazole ring), 7.57-7.31 (m, 4H, Ph), 7.25-6.94 (m, 4H, Ph-Pz ring), 5.23 (2H, s, CH_2), 4.01 (2H, s, CH_2), 3.34-3.07 (m, 8H, Piperazine ring); ^{13}CNMR (DMSO-*d*$_6$) δ(ppm): 168.2 (C=O), 157.5, 156.6, 143.8 (C-triazole), 141.4, 138.6, 126.9, 126.7, 125.7, 124.4, 123.1 (C-triazole), 120.4, 120.3, 118.5, 116.0, 115.8, 52.9, 50.3, 49.5; ESI-MS m/z: [M^++H] 463.18.

***2-(4-((4-(2-fluorophenyl)piperazin-1-yl)methyl)-1H-1,2,3-triazol-1-yl)-N-(3-(trifluoromethyl)phenyl)acetamide* (T8)**

Yield: 80%; Anal. Calc. For $C_{22}H_{22}F_4N_6O$: C 57.14, H 4.80, N 18.17%; found: C 57.20, H 4.85, N 18.25%; FTIR $\nu_{max}cm^{-1}$: 3182 (C-H triazole ring), 3084 (CH, Ar), 1725 (C=O), 1556 (C=C, Ar), 1340, 1315 (C-N), 1122, 1048 (C-C); ^{1}H NMR (DMSO-*d*$_6$) δ(ppm): 9.57 (s, 1H, NH), 7.76 (1H, s, triazole ring), 7.57-7.31 (m, 4H, Ph), 7.25-6.94 (m, 4H, Ph-Pz ring), 5.23 (2H, s, CH_2), 4.01 (2H, s, CH_2), 3.34-3.07 (m, 8H, Piperazine ring); ^{13}CNMR (DMSO-*d*$_6$) δ(ppm): 168.2 (C=O), 157.5, 156.6, 143.8 (C-triazole), 141.4, 138.6, 126.9, 126.7, 125.7, 124.4, 123.1 (C-triazole), 120.4, 120.3, 118.5, 116.0, 115.8, 52.9, 50.3, 49.5; ESI-MS m/z: [M^++H] 463.18.

***2-(4-((4-(2-fluorophenyl)piperazin-1-yl)methyl)-1H-1,2,3-triazol-1-yl)-N-(4-methoxyphenyl)acetamide* (T9)**

Yield: 82%; Anal. Calc. For $C_{22}H_{25}FN_6O_2$: C 62.25, H 5.94, N 19.80%; found: C 62.30, H 5.95, N 19.86%; FTIR $\nu_{max}cm^{-1}$: 3182 (C-H triazole ring), 3085 (CH, Ar), 1723 (C=O), 1565 (C=C, Ar), 1340, 1315 (C-N), 1120, 1050 (C-C); ^{1}H NMR (DMSO-d6) δ(ppm): 9.51 (s, 1H, NH),

8.33-8.31 (m, 2H, Ph), 7.76 (1H, s, triazole ring), 7.36-7.22 (m, 2H, Ph), 6.98-6.94 (m, 8H, Ph-Pz ring), 5.23 (2H, s, CH_2,), 4.01 (2H, s, CH_2), 3.80 (3H, s, CH_3), 3.34-3.07 (m, 4H, Piperazine ring); ^{13}CNMR (DMSO-d6) δ(ppm): 168.0 (C=O), 157.5, 156.4, 155.6, 143.8 (C-triazole), 141.4, 133.2, 124.4, 124.1, 123.3 (C-triazole), 122.2, 120.1, 118.5, 116.0, 115.8, 112.2, 55.3, 52.9, 52.7, 50.3, 49.5; ESI-MS m/z: [M^++H] 425.20.

2-(4-((4-(2-fluorophenyl)piperazin-1-yl)methyl)-1H-1,2,3-triazol-1-yl)-N-(4-nitrophenyl)acetamide **(T10)**

Yield: 82%; Anal. Calc. For $C_{21}H_{22}FN_7O_3$: C 57.40, H 5.05, N 22.31%; found: C 57.48, H 5.12, N 22.35%; FTIR ν_{max}cm^{-1}: 3180 (C-H triazole ring), 3085 (CH, Ar), 1725 (C=O), 1560 (C=C, Ar), 1340, 1315 (C-N), 1120, 1047 (C-C); ^{1}H NMR (DMSO-d_6) δ(ppm): 9.58 (s, 1H, NH), 8.21-7.79 (m, 2H, Ph), 8.21-7.79 (m, 2H, Ph), 7.77 (1H, s, triazole ring), 7.36-6.94 (m, 4H, Ph-Pz ring), 5.23 (2H, s, CH_2,), 4.01 (2H, s, CH_2), 3.34-3.07 (m, 8H, Piperazine ring); ^{13}CNMR (DMSO-d_6) δ(ppm): 168.2 (C=O), 157.5, 155.6, 143.8 (C-triazole), 143.2, 141.4, 125.1, 124.4, 124.1, 123.3 (C-triazole), 118.5, 118.0, 116.0, 52.9, 52.7, 50.2, 49.5; ESI-MS m/z: [M^++H] 440.18.

2-(4-((4-(2-fluorophenyl)piperazin-1-yl)methyl)-1H-1,2,3-triazol-1-yl)-N-(3-nitrophenyl)acetamide **(T11)**

Yield: 80%; Anal. Calc. For $C_{21}H_{22}FN_7O_3$: C 57.40, H 5.05, N 22.31%; found: C 57.48, H 5.12, N 22.35%; FTIR $\nu_{max}cm^{-1}$: 3185 (C-H triazole ring), 3085 (CH, Ar), 1723 (C=O), 1560 (C=C, Ar), 1340, 1315 (C-N), 1120, 1048 (C-C); ^{1}H NMR (DMSO-d6) δ(ppm): 9.56 (s, 1H, NH), 8.51-8.42 (m, 2H, Ph), 7.77 (1H, s, triazole ring), 7.74-7.56 (m, 2H, Ph), 7.36-6.94 (m, 4H, Ph-Pz ring), 5.23 (2H, s, CH_2,), 4.01 (2H, s, CH_2), 3.34-3.07 (m, 8H, Piperazine ring); ^{13}CNMR (DMSO-*d*$_6$) δ(ppm): 168.0 (C=O), 157.5, 155.6, 148.6 (C-triazole), 143.8, 141.4, 138.8, 130.3, 126.5, 124.4, 124.1, 123.3 (C-triazole), 118.5, 117.7, 116.0, 115.8, 114.5, 52.9, 52.7, 50.3, 49.5; ESI-MS m/z: [M^++H] 440.18.

N-(3,4-dichlorophenyl)-2-(4-((4-(2-fluorophenyl)piperazin-1-yl)methyl)-1H-1,2,3-triazol-1-yl)acetamide **(T12)**

Yield: 85%; Anal. Calc. For $C_{21}H_{21}Cl_2FN_6O$: C 54.44, H 4.57, N 18.14%; found: C 54.54, H 4.59, N 18.15%; FTIR $\nu_{max}cm^{-1}$: 3180 (C-H triazole ring), 3085 (CH, Ar), 1725 (C=O), 1560 (C=C, Ar), 1340, 1315 (C-N), 1120, 1048 (C-C); ^{1}H NMR (DMSO-d6) δ(ppm): 9.50 (s, 1H, NH), 7.88-7.87 (d, 1H, *J*= 5Hz, Ph), 7.77 (1H, s, triazole ring), 7.44-7.35 (m, 3H, Ph), 7.33-6.94 (m, 4H, Ph-Pz ring), 5.23 (2H, s, CH_2,), 4.01 (2H, s, CH_2), 3.34-3.07 (m, 4H, Piperazine ring); ^{13}CNMR (DMSO-d6) δ(ppm): 168.0 (C=O), 157.5, 155.6, 143.8 (C-triazole), 141.4, 136.4, 132.5, 129.7, 125.0, 124.4, 124.1, 123.3 (C-triazole), 122.2, 118.5, 116.0, 115.8, 52.9, 52.7, 50.3, 49.5; ESI-MS m/z: [M^++H] 463.11.
